# Reconciliation of titer differences between SARS-CoV-2 neutralizing antibody assays

**DOI:** 10.1128/jvi.00823-26

**Published:** 2026-08-27

**Authors:** Eva Stadler, Francesca Mordant, Matthew Gartner, Steffen S. Docken, Miles P. Davenport, David S. Khoury, Kanta Subbarao

**Affiliations:** 1 Infection Analytics Program, Kirby Institute, UNSW Sydney 2786, Sydney, New South Wales, Australia; 2 Department of Microbiology and Immunology, University of Melbourne, at the Peter Doherty Institute for Infection and Immunity, Parkville 534133, Melbourne, Victoria, Australia; 3 WHO Collaborating Centre for Reference and Research on Influenza, VIDRL at the Doherty Institute for Infection and Immunity, Melbourne, Victoria, Australia; St Jude Children's Research Hospital, Memphis, Tennessee, USA

**Keywords:** neutralization, assay differences, SARS-CoV-2, pseudovirus, microneutralization, plaque-reduction assay

## Abstract

SARS-CoV-2 antibody neutralization is a correlate of protection for COVID-19 and has been used to guide public health decisions. However, there is no standard neutralization assay, and absolute titers differ between assays. To investigate mechanisms causing these absolute titer differences, we compared 28 samples with three assays: a live virus cytopathic effect (CPE)-based microneutralization assay, a live virus focus reduction (immunospot, IS) assay, and a pseudotyped virus luciferase expression reduction (pseudovirus, PV) assay. We found significant correlations between all assays (ρ > 0.95 and *P* < 0.0001 for all pairwise comparisons), but also significant differences in absolute titers by assay (paired *t*-tests *P* < 0.001). IS and PV assays that assess neutralization as the titer required to reduce foci/luminescence by 50% had the smallest difference, with 1.2-fold higher IS assay titers (95% confidence interval, CI: 1.2–1.2). CPE assay titers were 5.5-fold lower (95% CI: 5.4–5.5) than in the IS assay. We show that these differences can be explained (i) by accounting for the size of inoculum being neutralized (half the infectious virions in the IS and PV assays, “per virion,” and the entire inoculum half the time in the CPE assay, “per well”) and (ii) by removing serum during the 5-day culture in the CPE assay (so serum can only neutralize the inoculum, rather than acting on the inoculum and slowing viral growth during culture). These findings highlight areas for more precise and harmonized terminology and particular assay elements that must be considered in efforts to harmonize neutralization assays.

**IMPORTANCE:**

Protection against COVID-19 is related to how well serum antibodies neutralize SARS-CoV-2. This neutralization can be assessed using different assays, which often provide significantly different estimates. To better understand why assay results vary, we compared three commonly used types of neutralization assay. We found both strong correlations and significant differences in measured neutralization between the different assays. Differences in the type of virus (live SARS-CoV-2 virus compared to a pseudotyped virus with SARS-CoV-2 spike protein), how long cells, virus, and antibody were allowed to interact, and whether neutralization of a batch of virions (“per well” neutralization) or individual infectious virions (“per virion”) was measured, all impacted virus neutralization. Understanding which factors have the largest impact on the differences between neutralization assays is an important first step toward harmonization of neutralization assays across the field.

## INTRODUCTION

Neutralizing antibody titers have been demonstrated to robustly and consistently correlate with protection from COVID-19 (1–4). Accordingly, measures of neutralizing antibody titers after vaccination have been considered in decisions on vaccine updates, as well as regulatory approvals (5–8). However, despite the well-established evidence that neutralizing antibodies correlate with protection, neutralization assays have not been standardized, and titers vary significantly between assays (9, 10). The neutralization assays used in the field differ in many aspects, including cell lines, viral strains, viral inoculum size, endpoints, and detection methods (11). It is sometimes suggested that some neutralization assays are superior to others. For example, it is commonly suggested that live virus assays (those using authentic SARS-CoV-2) are the gold-standard neutralization assay. Pseudotyped virus assays (those using a viral vector expressing SARS-CoV-2 spike proteins), while having advantages, need to be validated against live virus assays (10). In addition, it has been suggested that assays measuring cytopathic effect (CPE) are less consistent with other assays (12). Here, we consider the extent to which common neutralization assays are indeed similar or different by investigating absolute titer differences between assays. In particular, we aim to determine the extent to which different neutralization assays may or may not be fundamentally quantifying distinct biological processes, or the extent to which they simply capture the same processes with different sensitivity.

We directly test the factors that contribute to differences between three major assay types for assessing SARS-CoV-2 neutralization: a live virus CPE-based microneutralization (CPE) assay, a live virus focus (spot) reduction (immunospot, IS) assay, and a pseudotyped virus luciferase expression reduction (pseudovirus, PV) assay with replication-deficient lentivirus expressing SARS-CoV-2 Spike. Firstly, we assess differences in titers due to the use of live versus pseudotyped virus. Secondly, we examine the impact of other assay features in two different live virus assays: a focus reduction assay (IS) and a CPE microneutralization assay. These assays differ in that they measure either “per virion” neutralization (IS: the titer is the dilution that halves the number of spots, which are thought to be formed by a single infectious virion) or “per well” neutralization (CPE: the titer is the dilution that leads to infection, as evidenced by CPE, in half of the wells). Another difference is that the viral inoculum and serum are removed after 1 h of co-incubation on cells in the IS assay, whereas the viral inoculum and serum remain co-incubated with cells for the entire experiment in the CPE assay. This means that in principle the scoring of CPE may be impacted by antibodies’ neutralization of both the initial virus inoculum and subsequent virus growth in the CPE assay over 5 days.

Together, this work highlights that these three diverse neutralization assays all capture similar and highly related information, with major differences in absolute titers between each assay being explained by specific assay features.

## RESULTS

### Comparing live virus immunospot and pseudovirus luminescence reduction assays

To assess if neutralization titers differ based on use of an authentic SARS-CoV-2 (VIC01) or a pseudotyped virus expressing the spike protein of SARS-CoV-2, we assessed sera from 27 vaccinated subjects and a WHO International Standard plasma. The inclusion of the WHO positive International Standard plasma allowed us to normalize all estimated titers (ND50s) to IU/mL (Tables S1 to S5). Despite significant differences in absolute titers, we found high assay alignment after conversion to IU/mL (Fig. S7; Table S7), which supports the use of normalization to international units to reconcile titer differences for practical purposes. However, normalization does not provide insights into how assay design choices impact absolute titers in the IS, CPE, and PV assays (three different but commonly used neutralization assays). Thus, we investigated potential mechanisms that could cause absolute titer differences in these assays.

First, we compared assays with either the authentic live SARS-CoV-2 virus (immunospot, IS) or a replication-deficient lentivirus pseudotyped with SARS-CoV-2 S protein (pseudovirus, PV, Table 1) (two SARS-CoV-2-negative serum samples were also run as controls; their titers were undetectable and thus excluded from assay comparison). These assays both involve pre-incubation of virus with serial dilutions of serum, followed by placing the mixture onto cells to measure infectious non-neutralized virus. In the IS assay, the level of infection is detected by nucleocapsid staining and counting focus-forming units (“spots”) at 24 h. In the PV assay, infection level is detected by measuring total luminescence per well (in relative luminescence units, RLU) after infection with the pseudovirus carrying a nanoluciferase reporter gene (which is interpreted as a surrogate for the total number of infected cells). In each case, the neutralization titer is reported as the concentration of serum required to reduce the infection level (spot number or total luminescence) by 50%.

**TABLE 1:** Assay overview

	Cytopathic effect (CPE) assay	Immunospot (IS) assay	Pseudovirus (PV) assay
Virus	Authentic SARS-CoV-2 virus	Authentic SARS-CoV-2 virus	Lentivirus pseudotyped with SARS-CoV-2 S proteins
Cells	Vero E6-TMPRSS2 cells	Vero E6-TMPRSS2 cells	Vero E6-TMPRSS2 cells
Method	50 TCID_50_ virus is incubated with 1:2 serial dilutions of serum for 1 h Virus/serum mixture is transferred to cells and incubated for 5 days In the CPE assay with “inoculum removal,” the virus/serum mixture was washed twice and replaced with fresh media at 1 h CPE is scored by microscopy on day 5	Virus is diluted to 150–200 spots/well and incubated with 1:2 serial dilutions of serum for 1 h Virus/serum mixture is transferred to cells for 1 h Virus/serum mixture is replaced with a CMC overlay Plates are fixed after 24–26 h and stained for SARS-CoV-2 NP Foci are imaged and counted with a CTL scanner	Virus is diluted to 150,000 RLU/well and incubated with 1:2 serial dilutions of serum for 1 h Virus/serum mixture is transferred to cells On day 2, cells are lysed, luciferase substrate is added, and luminescence is measured on a plate reader
Readout	Cytopathic effect (CPE)	Focus-forming units (FFU)	Luciferase expression
Interpretation	Measures the dilution of serum that completely neutralizes all infectious virions in 50% of wells in a microplate: “**per well**” **neutralization**	Measures the ND50, i.e., the dilution of serum required to neutralize 50% of virus in each well: “**per virion**” **neutralization**	
	Measures both **neutralization of virus inoculum and impact on growth** until scoring of CPE. With “inoculum removal”: neutralization of virus inoculum only	Measures **neutralization of virus inoculum only** (due to overlay in IS assay and replication-deficient pseudotyped virus in PV assay)	

**Fig 1:**
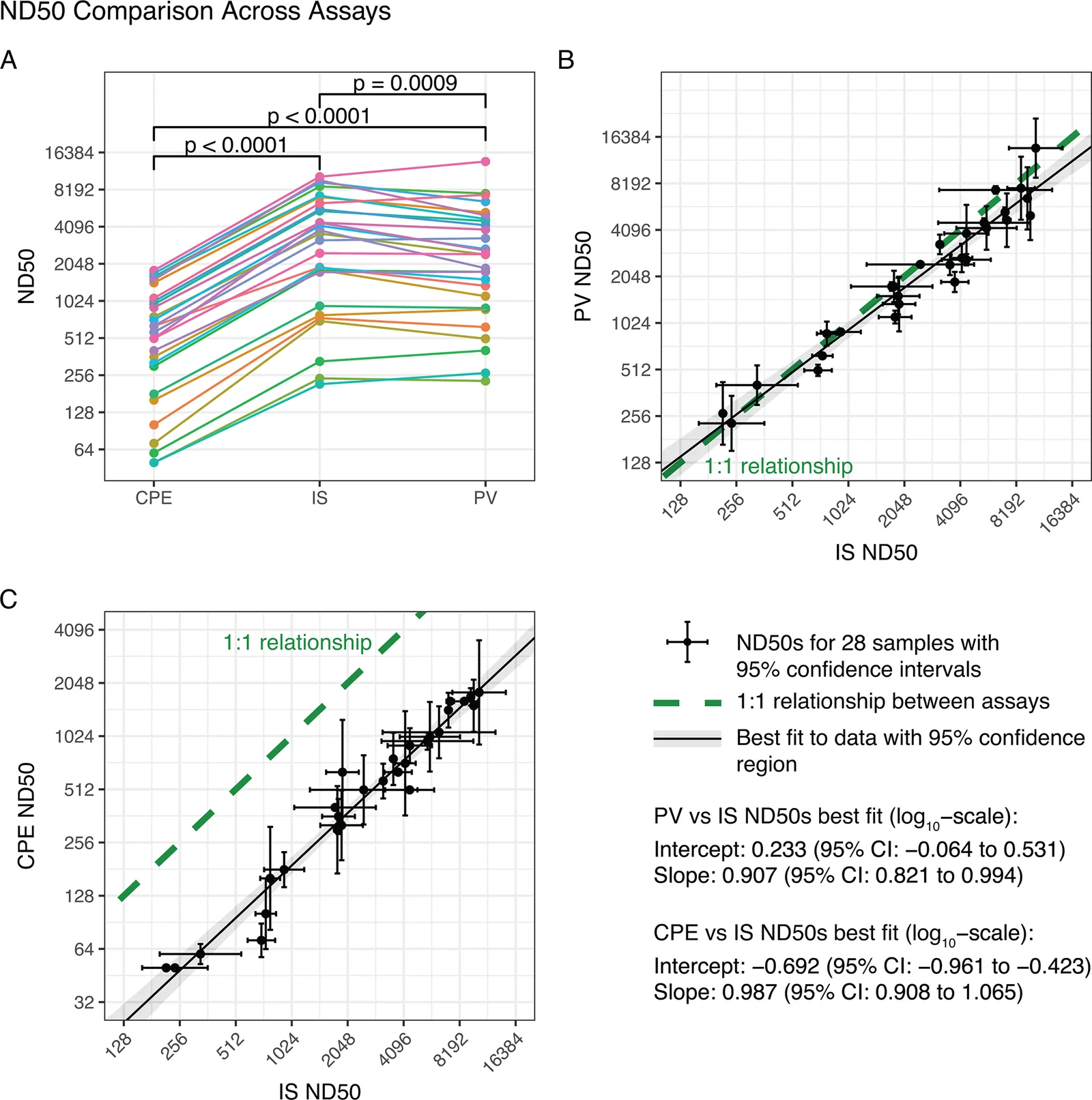
Assay comparison. (**A**) Comparison of neutralization titers (ND50s) for 28 samples assessed with the CPE, IS, and PV assay. Titers from the same sample are connected by a line, and log_10_ titers are compared using a paired *t*-test (*P* value indicated for all pairwise comparisons). (**B**) Comparison of PV (pseudotyped virus) versus IS (live virus) assays. Both assays are interpreted to measure “per virion” neutralization. (**C**) Comparison of neutralization assays assessing “per well” and “per virion” neutralization with live virus, CPE and IS assay, respectively. In panels B and C, the estimated titers for 28 samples are shown as black dots with 95% confidence bounds on the titers as black bars (see Tables S1 to S3). The best fit to the titers is shown as a black line with 95% confidence region as a gray shaded area. The green dashed line corresponds to a 1:1 relationship between the titers estimated with different assays, i.e., if titers agreed, they would fall on the green line.

Comparing the 50% neutralization titers between these assays, we found a strong correlation between them (ρ = 0.97, *P* < 0.0001, Spearman correlation; Fig. 1A and B; Tables S1 and S2). Even so, titers differed significantly (*P* = 0.0009, paired *t*-test) and were on average 1.2-fold higher with IS compared to PV (95% confidence interval, CI: 1.2–1.2). This may suggest that the authentic SARS-CoV-2 virus was more readily neutralized than the pseudotyped virus, or that halving luminescence in a well was not directly equivalent to halving the number of foci. However, this fold difference between IS and PV assay ND50s is within the variability between assay runs with either the IS or the PV assay (the average fold difference between assay runs was 1.3 for both the IS and PV assay). The very similar and highly correlated titers suggest that both assays are capturing very similar or the same information regarding the serum neutralizing capacity.

### Comparing live virus CPE microneutralization and immunospot assays

**Fig 2:**
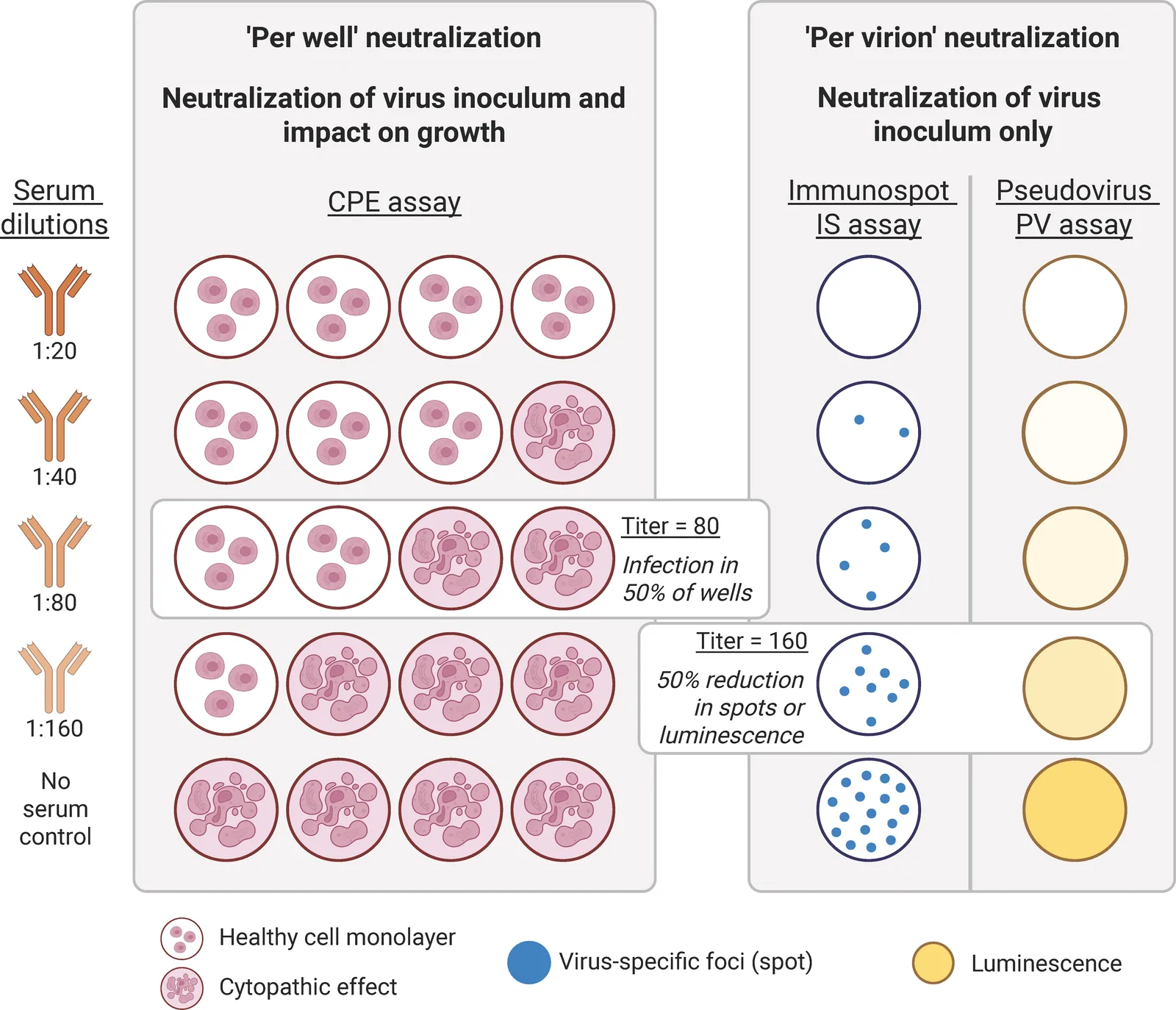
Schematic of the impact of “per well” neutralization (CPE assay, left) compared to “per virion” neutralization (IS and PV assays, middle and right, respectively). Serum at the dilution that neutralizes 50% of virions (1:160) leads to a 50% reduction in the number of spots and total luminescence (compared to a control without serum) in the IS or PV assay, respectively, i.e., this is the ND50 or neutralization titer. In the CPE assay, with 50% of virions neutralized, we expect residual virions to be present in the majority of wells and to cause infection (visible as cytopathic effect, CPE, by day 5, indicated by a red background for the well). Thus, more Ab is needed (lower dilution, 1:80) to neutralize 50% of wells, which leads to a lower estimated titer in the CPE assay due to the assay format of “per well” neutralization rather than “per virion” neutralization. Created in BioRender (Stadler E. 2026; https://BioRender.com/xg8kc99).

Next, we compared two different neutralization assays using the same authentic SARS-CoV-2 virus (VIC01) and cells (Vero E6-TMPRSS2 cells). The IS assay quantifies the serum titer required to reduce the number of foci by 50%, whereas the CPE quantifies the titer to prevent 50% of wells from forming CPE (cytopathic effect; Table 1). Even so, we found a strong correlation between estimated titers of the 28 samples (ρ = 0.98, *P* < 0.0001, Spearman correlation; Fig. 1C). However, there is a significant difference between titers, with on average 5.5-fold lower (“per well”) titers in the CPE assay compared to the IS (“per virion”) titers (95% CI: 5.4–5.5). On reflection, these differences seem reasonable, since the titer required to block half the number of foci can reasonably be interpreted as the titer required to block half the infectious virions, whereas blocking CPE formation in a well challenged with 50 TCID_50_ (50% tissue culture infectious dose) likely requires inhibiting more than half the infectious virions and may in fact require neutralizing all infectious virions in a well (Fig. 2).

### Modeling “per virion” and “per well” neutralization

**Fig 3:**
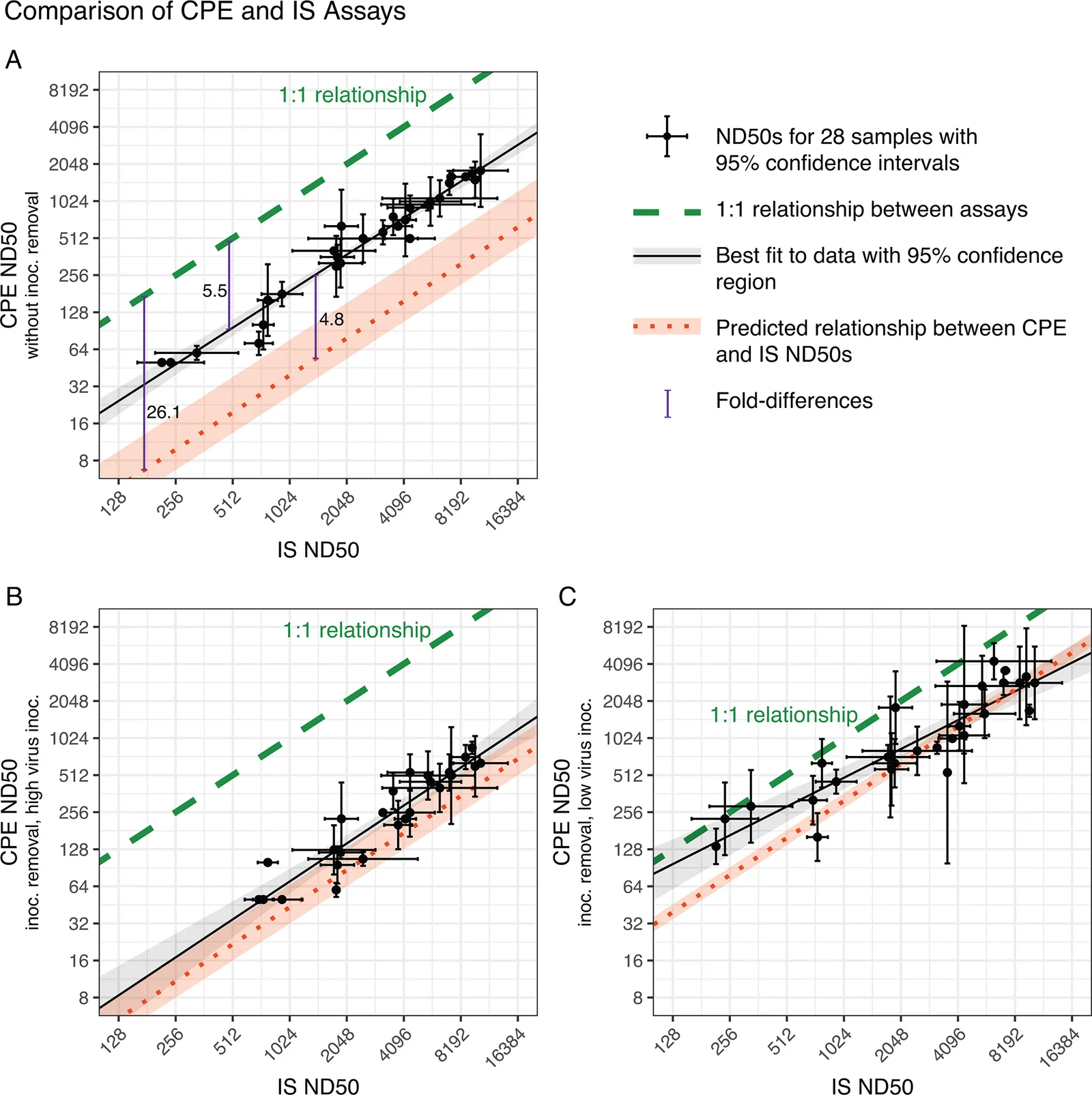
Comparison of CPE and IS assay titers and the impact of removing the inoculum in the CPE assay. (**A**) Comparison of CPE and IS assays with prediction of the assay difference based on assay format (“per well” vs “per virion” neutralization; red dotted line). The predicted difference between the CPE and IS assays was larger than the observed difference. This may be related to the persistence of serum in the culture preventing CPE formation. That is, in this CPE assay, the inoculum is not removed, i.e., serum can both neutralize the virus inoculum and impact viral growth for 5 days until scoring of CPE. (**B, C**) Comparison of IS assay with the CPE assay with inoculum removal (and two different virus inoculums). In these CPE assays and in the IS assay, the virus-serum inoculum is removed after a 1 h incubation (neutralization of virus inoculum only). Two different viral inoculums were used in the CPE assay: (**B**) 400 TCID_50_ and (**C**) 50 TCID_50_. These equated to an estimated 35.5 (95% CI: 21.4–49.5) and 3.3 (95% CI: 2.8–3.8) infectious virions per well in the two experiments, respectively (Table S8), as estimated from back titrations of the viral inoculum. In all panels, the estimated titers for 28 samples are shown as black dots with 95% confidence bounds on the titers as black bars (Tables S1 to S5). Three samples had undetectable ND50s in the CPE assay with inoculum removal and higher virus inoculum and are not shown (B, Table S5). The linear fit to the titers is shown as a black line with shaded 95% confidence region in gray. The green dashed line corresponds to a 1:1 relationship between the titers estimated with different assays, and the predicted relationship between assay titers is shown as a red dotted line (with shaded 95% confidence region). Slope and intercept for the linear fits and the predictions are in Tables S9.

As above, we note that the IS assay is interpreted as measuring the proportion of infectious virions neutralized at a given serum dilution (as evidenced by the number of spots, “per virion” neutralization). Whereas in the CPE assay, we added 50 TCID_50_ (estimated as 40 infectious virions per well using back-titration data, see Materials and Methods and Table S8) per well and measured the serum dilution that leads to CPE formation in 50% of wells where a single infectious virion is assumed to be able to cause evidence of CPE. We can use the neutralization curve from the IS assay to estimate the antibody titer required to neutralize the estimated 40 infectious virions in a well 50% of the time (Materials and Methods). This suggests that if we take the IS 50% (“per virion”) inhibitory titer, we expect a 26.1-fold lower titer (95% CI: 13.6 to 38.3) due to having to neutralize all 40 infectious virions in the CPE assay 50% of the time, i.e., simply due to the “per virion” or “per well” assay format (Fig. 2; see Materials and Methods; Table S9). However, although this comparison predicts titers should be 26.1-fold lower in the CPE assay compared to the IS assay, they are measured to be only 5.5-fold lower. Thus, the measured CPE titer is 4.8-fold higher than predicted (Fig. 3A), which might be explained by other differences between the assays.

### Duration of cell-virus-serum co-incubation impacts titers

The CPE and IS assays also differ in the duration for which virus, serum, and cells are in contact. In the IS assay, the virus-serum mixture is replaced with a carboxymethylcellulose (CMC) overlay after 1 h (effectively removing antibody). However, in the CPE assay, the virus-serum mixture and media remain in contact with the cells for 5 days of incubation. This longer exposure of cells to virus and serum could impact the estimated titers, as antibodies are present for longer, i.e., antibodies could appear more “potent” because antibodies may neutralize infectious virions in multiple rounds of infection and thereby reduce virus growth and prevent CPE formation. To investigate the effect of the duration of cell-virus-serum incubation, we repeated the CPE assay but included a new step of removing the virus-serum mixture after 1 h of incubation on cells (“inoculum removal,” Materials and Methods). Removing the inoculum reduced the estimated number of infectious virions per well from 40.0 (95% CI: 18.1 to 62.0) to 3.3 (95% CI: 2.8–3.8, Table S8)—which was estimated based on a back titration control and serum-free control wells (Materials and Methods). Thus, reducing the incubation time to 1 h lowers the effective virus inoculum approximately 12-fold. To account for this, we also ran this experiment with an increased virus inoculum of 400 TCID_50_ per well, which after 1 h of incubation and subsequent removal, led to an estimated 35.5 infectious virions per well (95% CI: 21.4–49.5). This is very similar to the estimated number of virions per well in the CPE assay without inoculum removal (i.e., 40.0 virions per well, Table S8).

From the above, we now have two equivalent CPE assays where the total effective inoculum equates to approximately 35–40 virions per well, but in one the inoculum is removed after 1 h and in the other it was not removed. We found that removal of the inoculum reduced titers by 2.6-fold (95% CI: 2.5–2.6) compared with when the serum was left on the culture (Fig. 3A and B; Fig. S6). This suggests that removing serum makes it more difficult to prevent CPE formation.

Having altered the CPE assay by removing inoculum after 1 h of incubation on cells, it now resembles the IS assay in the duration of cell-virus-serum co-incubation. Returning now to reconciling the CPE and IS assays, we see that the new CPE assay with removal of inoculum and the IS neutralization assays are again highly correlated (ρ = 0.94, *P* < 0.0001, Spearman correlation, Fig. 3B). We also find that the titers between this new CPE assay with serum removal were on average 14.2-fold (95% CI: 13.9–14.5) lower than in the IS assay, compared with the 5.5-fold difference between the original CPE assay and IS assay. Although the absolute titer difference between the assays is now bigger, the difference is similar to the expected difference of 23.6-fold (95% CI: 13.6–38.3), which is based on neutralization of 35.5 infectious virions per well (95% CI: 21.4–49.5, Tables S8 and S9) in the CPE assay compared to the “per virion” neutralization in the IS assay (Fig. 3B). Thus, absolute and predicted neutralization titers agree well for the CPE assay with removal of inoculum (even close to the assay’s limit of detection).

Finally, with inoculum removal in the CPE assay, the difference between “per virion” and “per well” neutralization titers should decrease with the number of infectious virions per well. Indeed, if there is just a single infectious virion per well, they should agree. To test this, we also compared CPE titers with inoculum removal but no increase in virus inoculum (i.e., where there were an estimated 3.3 infectious virions per well on average, 95% CI: 2.8–3.8) with IS “per virion” titers (Fig. 3C). Given the “per virion” neutralization titer from the IS assay and the estimated three infectious virions per well in the CPE assay (with 50 TCID_50_ and removal of the inoculum), we predict that the titer in the CPE assay should be 3.2-fold (95% CI: 2.8–3.7) lower than the IS assay. Indeed, we observe a 2.6-fold (95% CI: 2.5–2.7) difference between these assays (Fig. 3C). Together, these results suggest that as the number of infectious virions per well approaches 1, the “per well” assay and the “per virion” assays converge.

## DISCUSSION

*In vitro* neutralization assays are an important tool in assessing immunity to viral infection and were widely used in immunobridging of COVID-19 vaccines. There is currently no standardized assay for measuring SARS-CoV-2 neutralization *in vitro*, and individual assays can produce very different neutralization titers (13). We compared SARS-CoV-2 neutralization titers measured by three assays to investigate the potential mechanisms leading to differences in absolute titer measurements. The included assays differ in the viral construct (live vs pseudotyped virus) and the assay format (“per well” or “per virion” neutralization) and outcome (focus detection, luminescence reporter detection, and CPE). Titers were strongly correlated across the three assays. Comparing the IS and PV assays led to comparable titer estimates (PV titers were 1.2-fold lower, 95% CI: 1.2–1.2). This reflects the similarity of the endpoint being assessed in these assays, i.e., both attempt to quantify serum capacity to neutralize half of the infectious virions upon inoculation, despite obvious differences in the virus and the actual method of quantifying infection (a total per well luminescence vs counting foci). On the other hand, comparing two live virus assays (which used the same viral stock and cells), we found systematically lower titers with the CPE assay (CPE titers were 5.5-fold lower than IS titers, 95% CI: 5.4–5.5), but this difference could be explained based on two particular features of the assays. Firstly, the IS assay quantifies the titer required to neutralize half the infectious virions (counted as foci), whereas the CPE assay quantifies the titer required to prevent CPE formation in half of the wells (“per well” neutralization), which occurs by blocking 100% of a bolus of inoculum from infecting cells and replicating. This obviously requires more potent antibody neutralization, and thus the assay estimates lower titers. Secondly, since CPE formation requires ongoing viral replication and infection of cells, if antibody remains present during incubation of the cells with the viral inoculum, then it can reduce the probability of CPE formation. This has the impact of increasing the measured neutralization titers compared with when the serum is removed during the culturing step. By incorporating or accounting for these two attributes, the results of the IS assay could be harmonized with the results of the CPE assay. This is significant because identifying factors that distinguish neutralization assays highlights what each assay, in its native form, is quantifying. The IS assay appears to quantify the serum level required to block half of the virions in an infectious inoculum from infecting cells (*in vitro*), whereas the CPE assay measures the serum level required to block a bolus of virus from causing CPE by blocking the inoculum and/or blocking subsequent viral growth.

When using neutralization titers as a correlate of protection, immunity, or vaccine effect, then the “best” neutralization assay would be the one that most strongly predicts protection in clinical studies. Although it is not known which neutralization assay would be “best” correlated with protection, there are some factors that can be considered. Our analysis suggests that the number of infectious virions that need to be neutralized and the effects of continued exposure to antibody can affect the measured titers. *In vivo*, the size of the initial SARS-CoV-2 inoculum and the effects of antibody on subsequent viral proliferation are not well understood, i.e., it is not clear to which degree antibody acting to completely neutralize a viral inoculum at the site of entry (e.g., at a mucosal site) or antibody affecting subsequent viral replication kinetics (due to ongoing antibody effects) contributes to protection. However, both of these mechanisms may contribute to immunity and protection, and thus a neutralization assay that captures both blocking of the viral inoculum (with >1 infectious virion) and viral growth inhibition for multiple rounds of replication might be the most physiologically relevant. If this is the case, of the neutralization assays investigated here, this would most closely align with a CPE assay without removal of the virus and serum inoculum, but this cannot be confirmed without a clear understanding of clinical protection.

The strong correlation, good agreement with a linear relationship (on a log-log scale) between titers from different assays, and high alignment of titers after normalization, all suggest that these assays are likely equally suitable for defining a correlate of protection. The titer differences would lead to only a different scaling of the titer axis. However, an aggregation of titers from assays with different formats (without normalization and verification that normalization sufficiently reduces differences between assays) may introduce variability that can prevent or bias a correlation between neutralization titers and protection. While the effect of “per virion” and “per well” neutralization agreed well with a theoretical prediction and could therefore potentially be accounted for by a conversion factor, other effects such as different duration of cell-virus-serum incubation and factors not considered here are more challenging to correct for—even with normalization to IU/mL (14). For example, the duration of cell-virus-serum incubation may impact both the potency of antibodies and the number of infection events, which may further complicate attempts of reconciling assay differences (e.g., 40 vs 3 estimated infectious virions per well in the 5-day CPE assay without inoculum removal vs with inoculum removal after 1 h—with the same virus inoculum). Interestingly, although the viral construct (live or pseudovirus) and format (“per well” vs “per virion” neutralization) are typically reported, some factors impacting estimated titers (including duration of cell-virus-serum incubation) are inconsistently reported, which hinders attempts to account for these confounders.

Strengths of our approach included using the same cell lines, in the same laboratory, with the same authentic SARS-CoV-2 virus (in the two assays that used live virus), and the same samples across the different assays considered here. However, we have only investigated a relatively small sample panel (*n* = 28 serum samples) using three assays that were performed in the same laboratory. Thus, our conclusions may not be generalizable to monoclonal antibodies, across virus variants, or for assays performed in different laboratories with additional design differences that may cause titer differences. Other factors that may lead to differences in neutralization titers but were not considered here include cell lines, the specific pseudotyped virus (e.g., viral backbone), or other differences in assay protocols. For example, to assess differences between existing assays, we performed all assays using established protocols (as previously reported) (15, 16)—this included the use of TPCK trypsin in the CPE assay but not in the IS and PV assays, which may contribute to the residual differences between assays. In addition, there may be variability between different laboratories—even when using the same neutralization assay, which is not a factor we have considered here.

Practical considerations such as the cost, duration, high or low throughput, required materials or equipment, including viral isolates, and access to high-containment laboratories (if relevant) also affect the choice of a neutralization assay, and different assays may be most suitable for different purposes. None of the neutralization assays we assessed is a correct or incorrect choice; they merely measure the same biological process (neutralization of virions) in slightly different settings (e.g., neutralization of a batch of virions in a well compared to individual virions or impact of serum on only the first or multiple rounds of viral replication). However, to allow meaningful comparison and interpretation of neutralization titers across different assays, precision around terminology, with clear and complete reporting of assay parameters, such as the duration of cell-virus-serum incubation or the number of virions per well (for “per well” neutralization titers), is warranted.

## MATERIALS AND METHODS

### Neutralization assays

Authentic SARS-CoV-2 virus (hCoV/Australia/VIC01/2020, “VIC01”; GISAID: EPI_ISL_406844) was propagated in Vero E6-TMPRSS2 cells (Cell Bank Australia) and stored at −80°C.

For all antibody neutralization assays, 27 human serum samples were tested from individuals who had received either two or three doses of monovalent ancestral vaccine and that either did or did not have a breakthrough infection following vaccination. Additionally, positive international reference plasma obtained from NIBSC (sample 28 “NIBSC 21/338”) (17) was included in the analysis and used for titer normalization (Tables S1 to S5). Negative serum standards were below the limits of detection and thus excluded from titer comparisons, but they were used to estimate infectious virions per well in the CPE assay (see “Estimation of the number of infectious virions per well in the CPE assay,” below) and RLU per plate in the PV assay (Fig. S4). Sera were heat-inactivated at 56°C for 30 min. Each neutralization assay was run twice on different days, and reported dilutions are the dilutions of samples after virus was added.

### Cytopathic effect (CPE) assay

Virus was titrated by performing serial 10-fold dilutions on Vero E6-TMPRSS2 cell monolayers cultured in 96-well plates, and cytopathic effect was read on day 4 to calculate the TCID_50_ using the Reed-Muench method. Samples were serially diluted twofold and incubated with 50 TCID_50_ for 1 h. Virus/serum mixture was transferred to monolayers of Vero E6-TMPRSS2 cells in DMEM media containing 1 µg/mL TPCK trypsin (Worthington, #LS003740) that had been seeded in 96-well plates 2 days prior, in quadruplicate, and cells were incubated at 37°C/5% CO_2_. CPE was read on day 5, and titers were calculated for each assay run using the Reed-Muench method. The ND50s (and 95% CIs) were calculated as the geometric mean of ND50s for the two assay runs (Tables S3 to S5). If more than 50% (>2 out of 4 wells) were positive for CPE at the lowest dilution, then the titer was below the limit of detection.

### CPE assay with inoculum removal

The CPE assay was performed as described above. However, the virus/serum mixture was incubated on Vero E6-TMPRSS2 monolayers for 1 h before being removed, washed once with plain DMEM media, which was replaced again with plain DMEM media containing 1 µg/mL TPCK trypsin.

### Immunospot (IS) assay

SARS-CoV-2 VIC01 virus was titrated by serially diluting the virus threefold and transferring the dilution series to monolayers of Vero E6-TMPRSS2 cells, which were then incubated together for 1 h. The supernatant was removed and replaced with a final concentration of 1% CMC (Sigma-Aldrich, #C4888-500G) overlay mixed in equal parts with 2× DMEM media (made in-house). Plates were incubated at 37°C for 24 h, then fixed with 10% formalin overnight and washed six times with PBS before proceeding with immunostaining steps described below. We selected a dilution of the virus inoculum to ensure that the IS assay was in its linear dynamic range, i.e., a 50% reduction in the focus (spot) count corresponds to a 50% reduction in the virus inoculum and reduces the chances of foci merging (Fig. S1). The virus inoculum was diluted to a concentration of 200 foci/well for all runs of the IS assay.

To perform the IS antibody neutralization assay, sera were serially diluted twofold and incubated for 1 h with an equal measurement of diluted virus. Media was removed from monolayers of Vero E6-TMPRSS2 cells in 96-well plates, and serum-virus mixture was added to cells and incubated at 37°C for 1 h. Serum-virus mixture was removed and replaced with a final concentration of 1% CMC overlay mixed in equal parts with 2× DMEM media. Plates were incubated at 37°C for 24 h, fixed with 10% formalin overnight, and washed with PBS six times before immunostaining.

For immunostaining, all steps were performed at room temperature. Wells were treated with 0.2% (vol/vol) Triton X-100 (Sigma-Aldrich, #X100-100ML) in PBS for 10 min, then washed twice with PBS. Wells were treated with 0.3% (vol/vol) hydrogen peroxide (Merck, #H1009) in distilled water for 20 min, then washed twice with PBS. Anti-SARS-CoV-2 nucleocapsid rabbit polyclonal antibody (Sino Biologicals, #40588-T62) was diluted 1:1,000 in 0.2% (vol/vol) Triton X-100 in PBS and incubated on plates for 1 h before being washed twice with PBS. Anti-rabbit IgG goat-horseradish peroxidase conjugate (Cell Signaling Technology, #7074S) was diluted 1:2,000 in PBS and incubated on wells for 1 h before being washed twice with PBS. TrueBlue Peroxidase Substrate (KPL #5510-0050) was incubated on plates for 10 min, then plates were washed four times with distilled water and dried overnight. Plates were imaged using an ImmunoSpot S6 Ultra-V analyzer (Cellular Technologies Europe), and foci were counted using the BioSpot counting module.

### Pseudovirus (PV) assay

To generate SARS-CoV-2 pseudoviruses, lentiviral transfer plasmid HRsin-MK2-nLUC (generated by Prof. Melissa Churchill), lentiviral packaging plasmid psPAX2, and the pcDNA3.1 encoding SARS-CoV-2 (Wuhan-1) Spike protein (18) were used. Specifically, HEK293T cells were seeded into a T75 flask 1 day prior to transfection in DMEM media containing 10% fetal bovine serum (FBS) without antibiotics. A total of 15 µg HRsin-MK2-nLUC, 10 µg psPAX2, and 5 µg SARS-CoV-2 Wuhan-1 were mixed with Opti-MEM (Gibco, #31985070) and Lipofectamine 2000 (Invitrogen, #11668019) transfection reagent for 25 min and incubated with cells for 6 h at 37°C before medium was replaced with DMEM containing 10% FBS and penicillin-streptomycin (Gibco, #15140122), and then incubated again at 37°C for 2 days. Supernatant containing pseudovirus was collected, centrifuged at 500 *× g* for 5 min, filtered through a 0.45 µm syringe filter, aliquoted, and stored at −80°C.

The PV assay uses pseudoviruses with the Wuhan-1 Spike (NC_045512.2), while the authentic virus used in the IS and CPE assays is VIC01 (MT007544.1). Between the spikes of the pseudoviruses and authentic virus, there are three changes: (i) S247R within the N-terminal domain, (ii) H655Y near the furin cleavage site (this change came up during VIC01 stock during passage), and (iii) the pseudovirus has an 18 amino acid truncation at the C-terminal domain to improve incorporation into the lentiviral backbone.

Pseudoviruses were titrated by using 10-fold serial dilutions on monolayers of Vero E6-TMPRSS2 cells seeded the day prior in white 96-well plates. Luciferase RLU were measured as described below to identify the dilution corresponding to 150,000 RLU/well and to ensure that the PV assay was in its linear dynamic range, i.e., a 50% reduction in RLU corresponds to a 50% reduction in the virus inoculum (Fig. S2).

For PV neutralization assays, sera were serially diluted twofold, mixed in equal parts with a dilution of pseudovirus corresponding to 150,000 RLU/well, and incubated at 37°C for 1 h. Media were removed from monolayers of Vero E6-TMPRSS2 cells seeded the day before, the serum-virus mixture was transferred to cells, and plates were incubated at 37°C for 2 days.

To measure luciferase expression, supernatant was removed from cells, and 1× Passive Lysis Buffer (Promega, #E1941) was incubated on cells at room temperature for 10 min. Luminescence signal was measured using the Nano-Glo Luciferase Assay System (Promega, #N1130), and RLU values were obtained using a FLUOstar Omega plate reader (BMG Labtech).

### Estimation of neutralization titers

For the IS and PV assays, neutralization titers for all samples were estimated as the ND50 (dilution that reduces spots or luminescence by 50% compared to no serum controls) by fitting a logistic function to the data:


(Eq. 1) $$logistic(d|m,k,ND50)=\frac{m}{1+\exp\left(-k\times \left(\log_{10}(d)-\log_{10}(ND50)\right)\right)},$$

where *d* is the dilution, *m* is the maximal value of the logistic function (maximal number of spots for the IS assay and maximal RLU in the PV assay), *k* is the slope (the same for all samples, but assay repeats were fitted separately), and ND50 is the neutralization titer. Both the slope (*k*) and the titer (ND50) were log-transformed for fitting; the maximum of the logistic function (*m*) was not log-transformed (linear scale).

We used a least squares approach to fit a logistic function to the number of spots or RLU by dilution, with the maximal number of spots or luminescence estimated simultaneously by fitting to controls on the same plate (Fig. S3 and S4). Assay runs were analyzed separately, and the ND50s (and 95% CIs) were calculated as the geometric mean of ND50s for the two independent assay runs (Tables S1 and S2). If the estimated titer for a sample was below the lowest dilution used in either assay repeat, then the titer was below the limit of detection. For the IS and PV assays, the estimated titer was above the lowest dilution in one or both assay repeats for all samples, i.e., all samples had detectable neutralization titers. All model fitting and statistical analyses were performed in R (version 4.3.2). Statistical analyses were conducted at a significance level of 0.05, and all confidence intervals (CIs) are 95% CIs.

### Estimation of the number of infectious virions per well in the CPE assay

The number of infectious virions per well in the CPE assay is important in reconciling differences between the IS (“per virion”) assay and the CPE (“per well”) assay, especially in the CPE assays with removal of the inoculum (since the TCID_50_ inoculum does not reflect the effective number of infected wells observed). To estimate the number of infectious virions per well in this case, we used back titration data, virus-only control data, SARS-CoV-2 negative control sera, and a maximum likelihood approach.

We assume that the number of infectious virions per well is Poisson distributed, and a well is negative if there are no infectious virions in that well, i.e., for a well receiving an undiluted inoculum,


(Eq. 2) $$P(\text{negative well})=P(0\;\text{virions in the well})=Pois(0|n_{vir})=e^{-n_{vir}}$$

where *n*_vir_ is the average number of infectious virions in a well. Thus, we minimized the negative log-likelihood function


(Eq. 3) $$l(n_{vir})=-\sum_{\begin{array}{c} \text{sample }s,\\ \text{dilution }d \end{array}}\log\left(Binom\left(x={neg\;wells}_{s,d},n={total\;wells}_{s,d},p=\exp(-d\times n_{vir})\right)\right),$$

where the sample *s* refers to different data (back titration, virus-only control, or SARS-CoV-2-negative control sera), *d* is the dilution factor for the virus inoculum, Binom(*x*, *n*, *p*) is the probability mass function of the binomial distribution (with *x* successes, *n* trials, and success probability *p* for each trial), and neg wells*_s,d_* and total wells*_s,d_* are the number of negative wells and total number of wells for sample *s* and dilution *d*, respectively.

### Assay comparison

We compared absolute titers for the same 28 samples measured with the CPE, IS, and PV assay (Tables S1 to S5). Samples with non-measurable titers (below the limit of detection) were excluded from assay comparisons. We compared neutralization titers from different assays using paired two-tailed *t*-tests of log_10_-transformed titers (Fig. 1A) and Spearman’s rank correlation. Fold differences between neutralization titers were calculated as the geometric mean of fold differences between titers of the same sample with 95% CI of the geometric mean of fold differences. For visual comparisons of neutralization titers, we show the titers with one assay against the titer of the same sample with another assay (with titers on a log-scale; Fig. 1B, C and 3). We also fitted a linear regression to show the best linear fit to the titers (shown in figures with a black line and shaded gray 95% confidence region).

### Prediction of CPE “per well” ND50s based on IS “per virion” ND50s

Here, we derive a relationship between CPE and the IS ND50s. For a given sample, we assume that the logistic function fitted to the IS data represents the probability of neutralizing a particular infectious virion at each dilution of the sample. From this, we can then calculate the associated probability to neutralize a given number of infectious virions (*n*). That is, if the probability of neutralizing any particular virion is *p*, then the probability of neutralizing all *n* infectious virions is *p^n^*. From this, we can estimate the relationship between the ND50 in the IS assay and the ND50 in a CPE assay (where a well will be positive or negative depending on whether all of the inoculum placed in a well is neutralized). The details of this derivation can be found in the supplemental material. With this approach, we find the following predicted relationship between CPE “per well” ND50 and the IS “per virion” ND50:


(Eq. 4) $$\log_{10}(ND50_{CPE})=\log_{10}\;(ND50_{IS})+\frac{1}{k}\times \log\;\left(\frac{1}{2^{n}}-1\right),$$

where ND50_CPE_ and ND50_IS_ are the neutralization titers estimated with the CPE and IS assays, respectively; *k* is the slope of the logistic function for the IS assay; and *n* is the estimated number of infectious virions in a well in the CPE assay. We estimated the uncertainty of the predicted CPE titer by bootstrapping: we sampled 100,000 slopes and number of infectious virions (using the variance from the slope fitted to the first and second run of the IS assay and estimating the number of infectious virions from control data, respectively) and calculated the 2.5th and 97.5th percentiles to obtain 95% CIs.

In visual comparisons of the CPE and the IS neutralization titers, we have added this prediction with the 95% confidence region to show the theoretically expected relationship between the titers based on the “per well” and “per virion” assay format (red dotted line with red shaded 95% confidence region; Fig. 3).

## Data Availability

Data and code are publicly available at https://doi.org/10.5281/zenodo.21845043.

## SUPPLEMENTAL MATERIAL

Supplemental material jvi.00823-26-s0001.pdf Fig. S1 to S7; Tables S1 to S9.

ASM does not own the copyrights to Supplemental Material that may be linked to, or accessed through, an article. The authors have granted ASM a non-exclusive, world-wide license to publish the Supplemental Material files. Please contact the corresponding author directly for reuse.
