# Supplementary material for: Reconciliation of titer differences between SARS-CoV-2 neutralizing antibody assays
Source: J Virol. 2026 Aug 27;100(9):e00823-26. doi: 10.1128/jvi.00823-26 (PMC13595964; doi:10.1128/jvi.00823-26)
Supplement: Supplementary file 1 [file jvi.00823-26-s0001.pdf]

Reconciliation of titer differences between SARS-CoV-2 neutralizing antibody assays

Appendix

Eva Stadler,* Francesca Mordant,* Matthew Gartner, Steffen S. Docken, Miles P. Davenport,‡ David S. Khoury,‡ Kanta Subbarao‡#

# Address correspondence to Kanta Subbarao, kanta.subbarao@unimelb.edu.au.
*,‡ These authors contributed equally.

Table of contents

Methods 2

Derivation of the prediction of CPE titers based on IS titers 2

Figures 4

Fig. A 1 IS assay viral inoculum quality control 4
Fig. A 2 PV assay pseudovirus inoculum quality control 5
Fig. A 3 Fit to the IS assay data 6
Fig. A 4 Fit to PV assay data 7
Fig. A 5 CPE ND50 comparison with and without removal of inoculum 8
Fig. A 6 CPE ND50 comparison with and without removal of inoculum (comparable estimated number of infectious virions per well) 9
Fig. A 7 Comparison of normalized ND50s measured with IS, PV, and CPE assay 10

Tables 11

Table A 1 IS assay: Estimated ND50s 11
Table A 2 PV assay: Estimated ND50s 12
Table A 3 CPE assay: Estimated ND50s 13
Table A 4 CPE assay (with inoculum removal, lower virus inoculum): Estimated ND50s 14
Table A 5 CPE assay (with inoculum removal, higher virus inoculum): Estimated ND50s 15
Table A 6 Mean fold-difference of ND50s between assays 16
Table A 7 Mean fold-difference of normalized ND50s between assays 16
Table A 8 Estimated number of infectious virions per well in CPE assays 17
Table A 9 Observed and predicted relationships between CPE and IS assay ND50s 17

Methods

Derivation of the prediction of CPE titers based on IS titers

In the Immunospot (IS) assay, the number of 'spots' or foci at each dilution of serum are quantified for each sample (n=27 serum samples and 1 control). To estimate the neutralization titer (ND50) associated with a 50% reduction in the number of foci, we fit a (3 parameter) logistic function relating the number of foci to the serum dilution (Materials and Methods, Eq. 1). Normalizing this relationship by the average number of foci in the absence of neutralization, we derive a function that can be interpreted as the probability that a virion is not neutralized at dilution d:

$$P(1 \text{ virion not neutralized} \mid \text{dilution } d) = \text{logistic}(d \mid m, k, ND50)/m, \qquad \text{(Eq. A 1)}$$

where d is the dilution, 'logistic' is the logistic function defined in (Eq. 1), m is the average number of foci in the absence of neutralization (the maximum of the logistic function), k is the slope (average over assay repeats), and $ND50$ is the neutralization titer. Note that 'virions' means 'infectious virions' here and we omit 'infectious' for the sake of brevity.

The IS assay measures 'per virion' neutralization whereas the CPE assay measures 'per well' neutralization. For a well in the CPE assay to be negative (i.e. show no cytopathic effect (CPE)), we assume that all infectious virions in that well must be neutralized. Thus, instead of describing the probability of a single virion being neutralized (like the IS assay), it describes the probability of neutralizing n virions, where n is the number of virions in a well.

With this interpretation, the CPE ND50 ($ND50_{CPE}$) is the serum dilution at which n virions are neutralized in 50% of the wells, i.e. at the CPE ND50 n virions (within 1 well) are neutralized 50% of the time, which is given by

$$P(n \text{ virions neutralized} \mid d = ND50_{CPE}) = 0.5. \qquad \text{(Eq. A 2)}$$

We assume that virions are independent (i.e. neutralization of one virion does not affect another virion), then we can re-write (Eq. A 2) as:

$$\left(P(1 \text{ virion neutralized} \mid d = ND50_{CPE})\right)^n = 0.5. \qquad \text{(Eq. A 3)}$$

Given our above description of the IS assay as providing the probability that any particular infectious virion is not neutralized (thus causing formation of a foci), we see that (Eq. A 3) can be written as:

$$\left(1 - P(1 \text{ virion not neutralized} \mid d = ND50_{CPE})\right)^n = 0.5. \qquad \text{(Eq. A 4)}$$

Combining (Eq. A 4) with (Eq. A 1) and (Eq. 1), we find:

$$\left(1 - \frac{1}{1 + \exp\left(-k \times \left(\log_{10}(ND50_{CPE}) - \log_{10}(ND50_{IS})\right)\right)}\right)^n = 0.5, \qquad \text{(Eq. A 5)}$$

where $ND50_{CPE}$ and $ND50_{IS}$ are the neutralization titers with the CPE and IS assay, respectively. This equation relates the CPE ND50 to the IS ND50, taking as additional inputs the slope of the logistic curve for the IS assay (k) and the estimated number of infectious virions in a well in the CPE assay (n). Re-arranging the terms in (Eq. A 5), we find that

$$\log_{10}(ND50_{CPE}) = \log_{10}(ND50_{IS}) + \frac{1}{k} \times \log\left(2^{\frac{1}{n}} - 1\right), \qquad \text{(Eq. A 6)}$$

where ‘log’ is the natural logarithm.

Figures

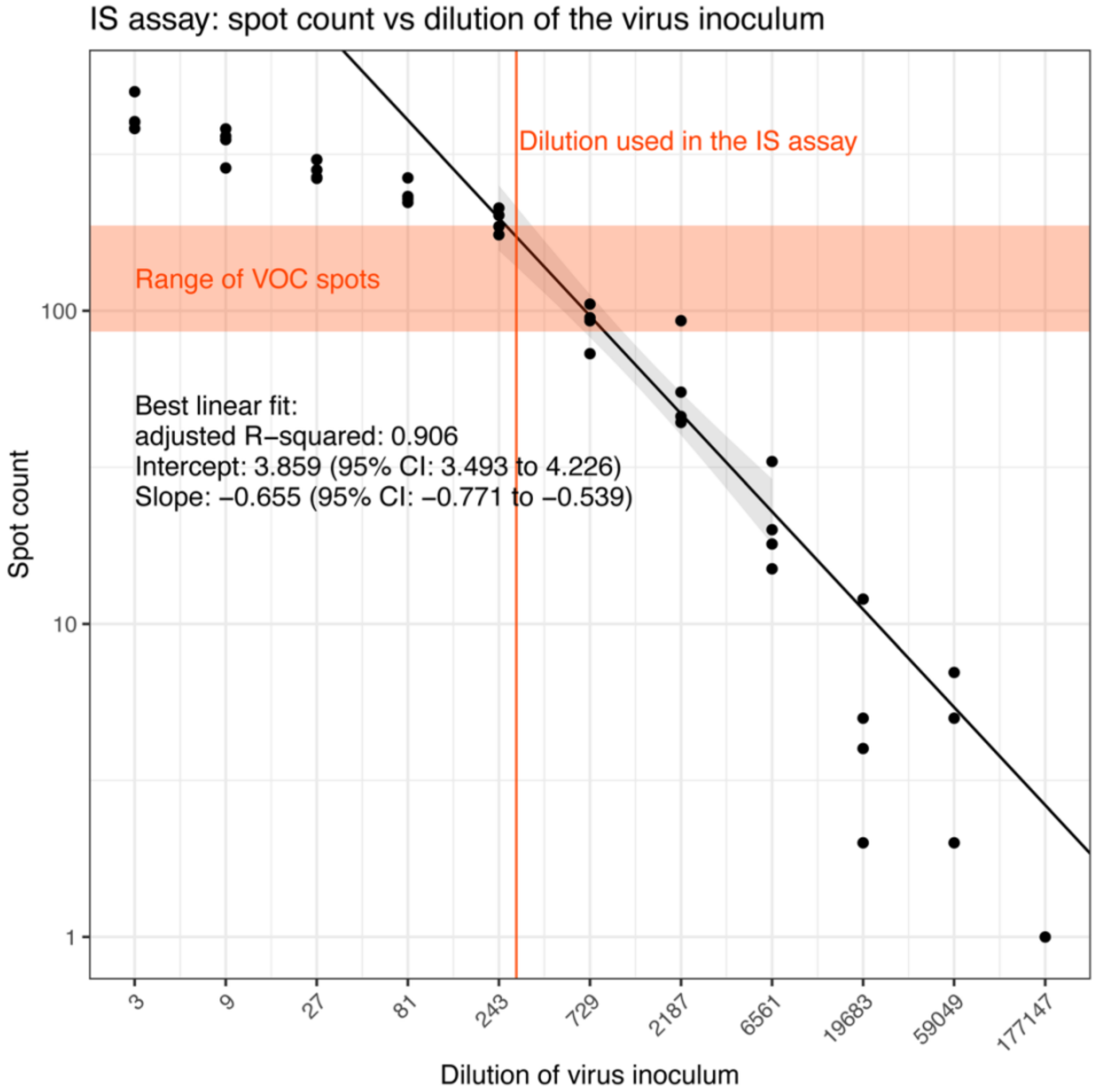

Fig. A 1 IS assay viral inoculum quality control

Spot count by dilution of virus inoculum. The black points show the data (3 data points with 0 spots for dilution 177,147 were excluded due to the log_{10}-scale of the spot count). The black line and grey shaded area show the linear fit on a log_{10}-log_{10}-scale (as shown) where the data range from dilution 243 to 6561 was selected based on the adjusted R^2 (shown with parameters of the fit in black text). The dilution used in the final runs of the IS assay was 1:300 and is indicated by a red vertical line. The orange shaded region shows the spot count range for the VOC (virus only control) samples in both final IS assay runs (ND50s reported in Table A 1). Thus, the viral inoculum used in this assay is in the linear range of spots (focus forming units), and thus foci merging or overlap is unlikely to have a major impact on the number of foci.

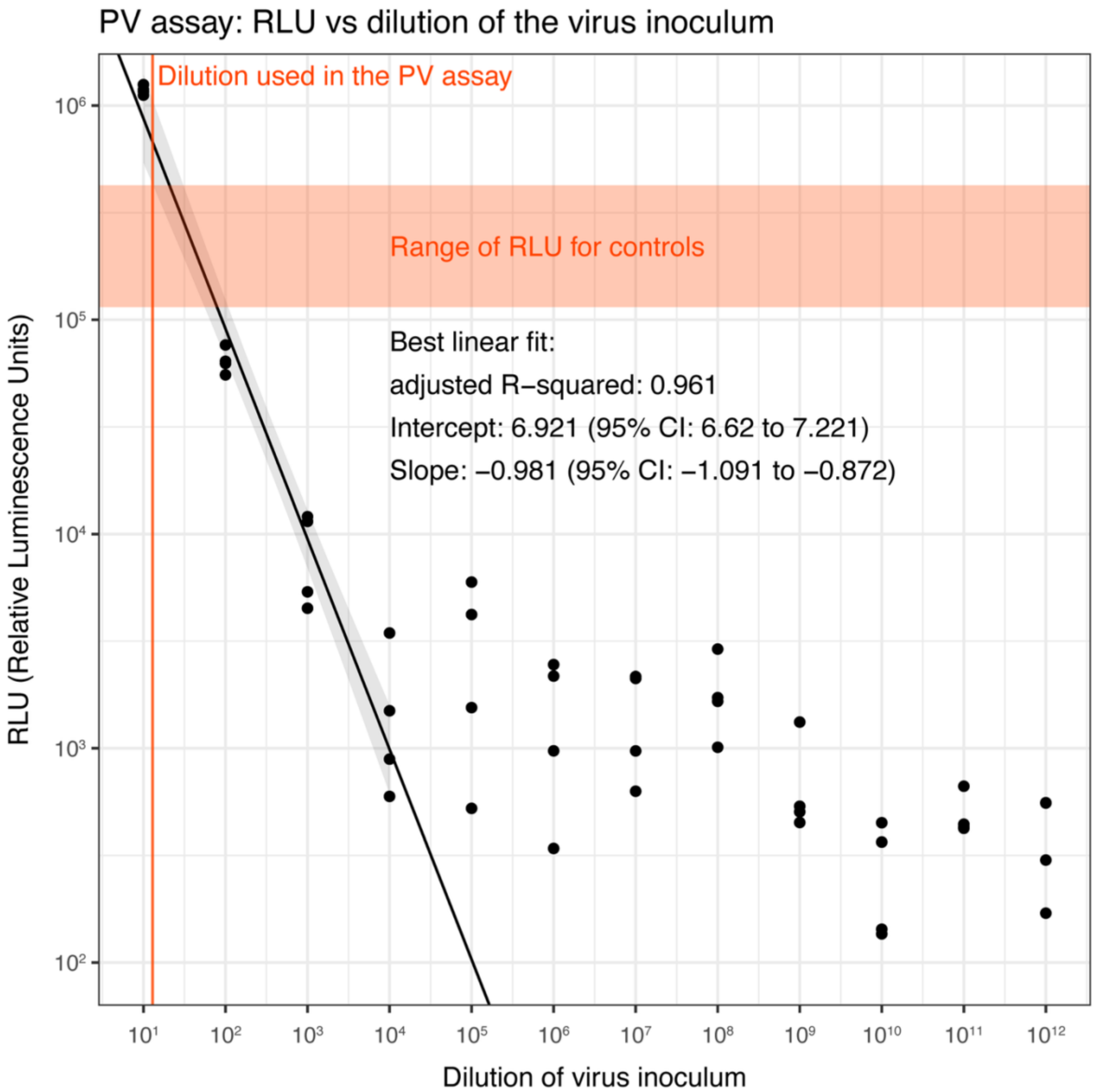

Fig. A 2 PV assay pseudovirus inoculum quality control

RLU by dilution of virus inoculum. The black points show the data. The black line and grey shaded area show the linear fit on a $\log_{10}$-$\log_{10}$-scale (as shown) where the data range from dilution 10 to 10^4 was selected based on the adjusted R^2 (shown with parameters of the fit in black text). The dilution used in the final runs of the PV assay was 1:12.9 and is indicated by a vertical red line. The orange shaded region shows the RLU range for virus control samples in both final PV assay runs (ND50s reported in Table A 2). Thus, the virus inoculum used in the PV assay falls within the assay's linear dynamic range.

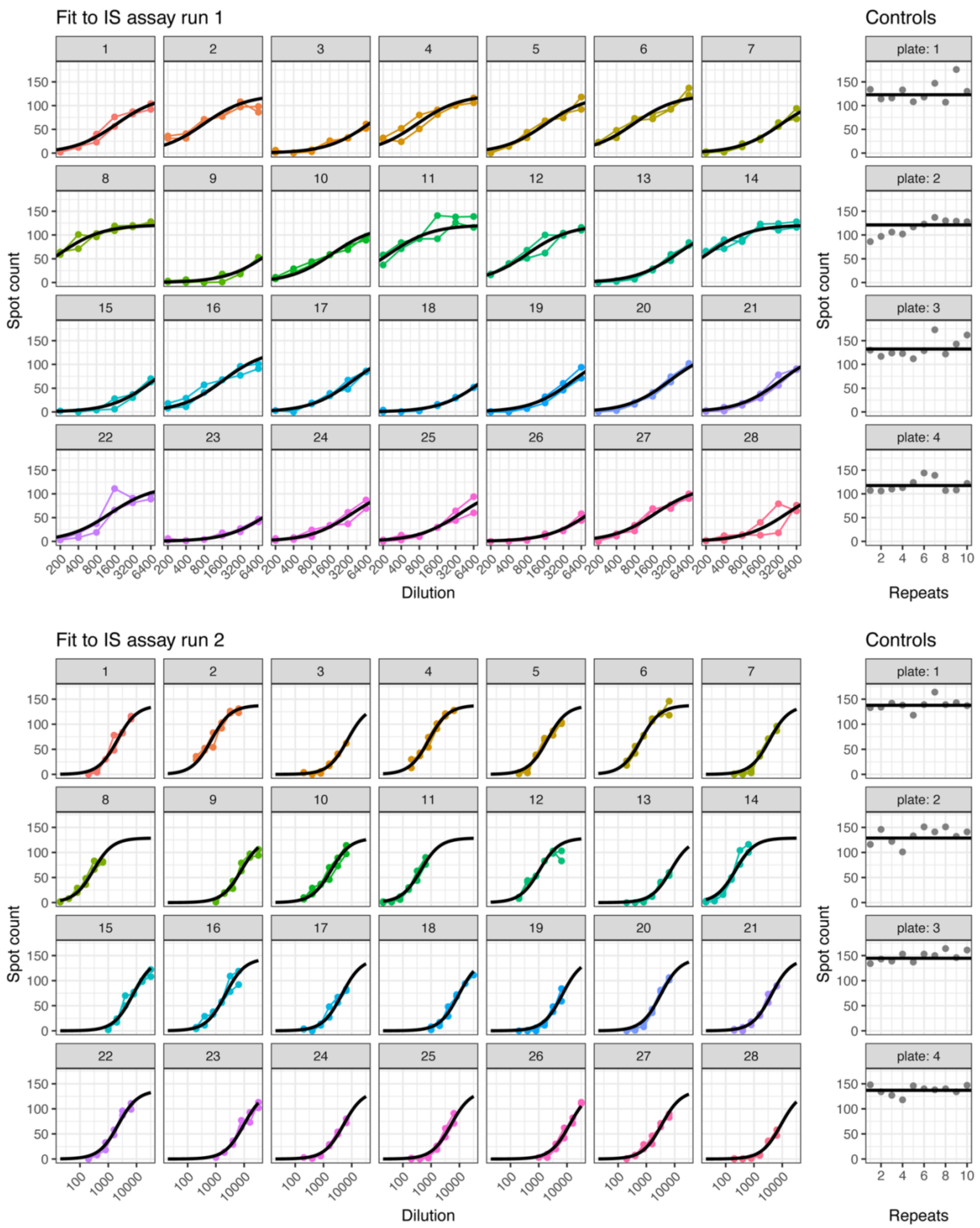

Fig. A 3 Fit to the IS assay data

Each of the two IS assay runs was fitted separately to estimate ND50s for each sample in each run (Table A 1). The maximum number of spots per plate and assay run was fitted simultaneously (samples shown in the same row were on the same plate, controls are virus-only control).

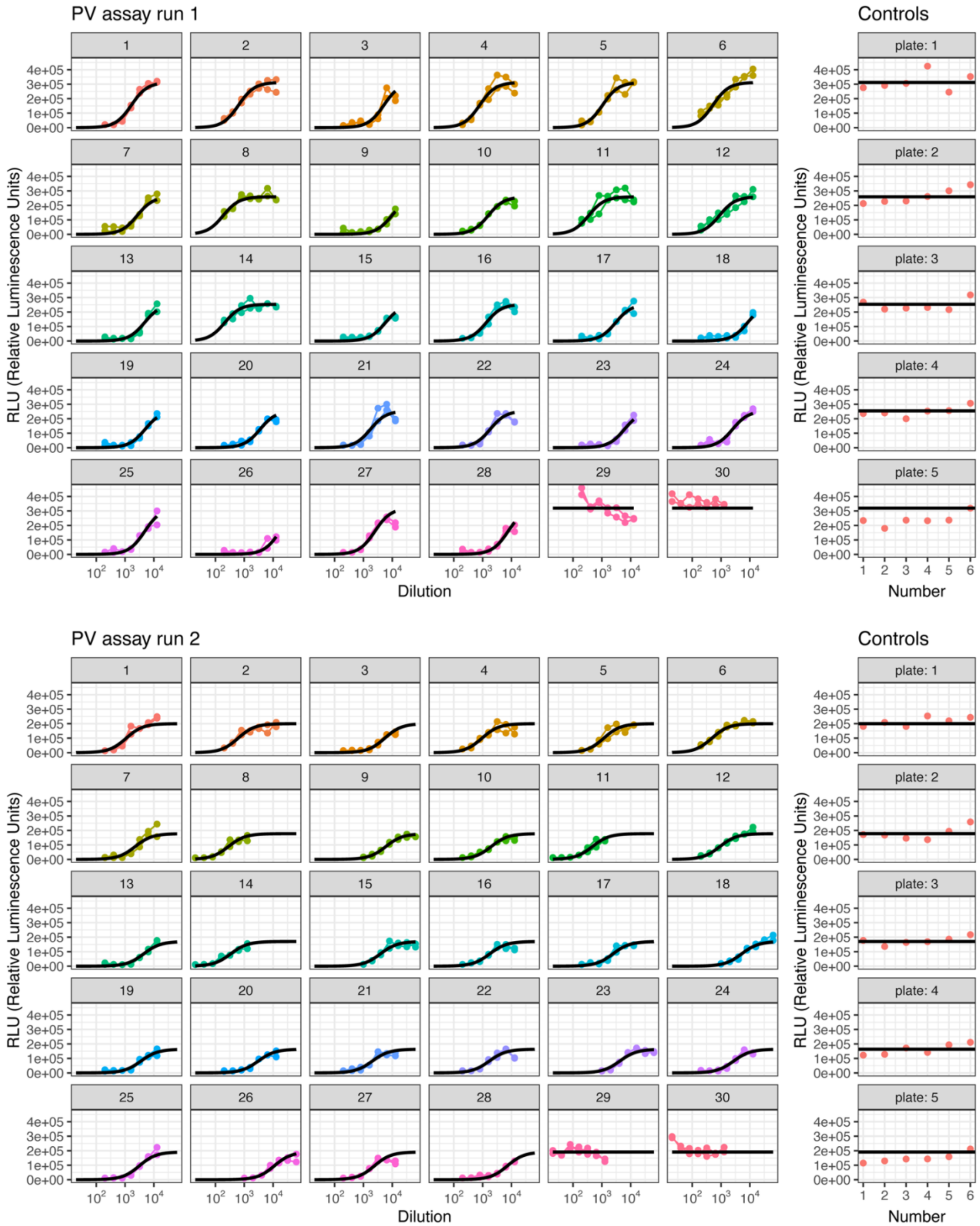

Fig. A 4 Fit to PV assay data

Each of the two PV assay runs was fitted separately to estimate ND50s for each sample in each run (Table A 2). The maximal RLU per plate and assay run was fitted simultaneously (samples shown in the same row were on the same plate). Note that samples 29 and 30 were additional negative control samples on plate 5.

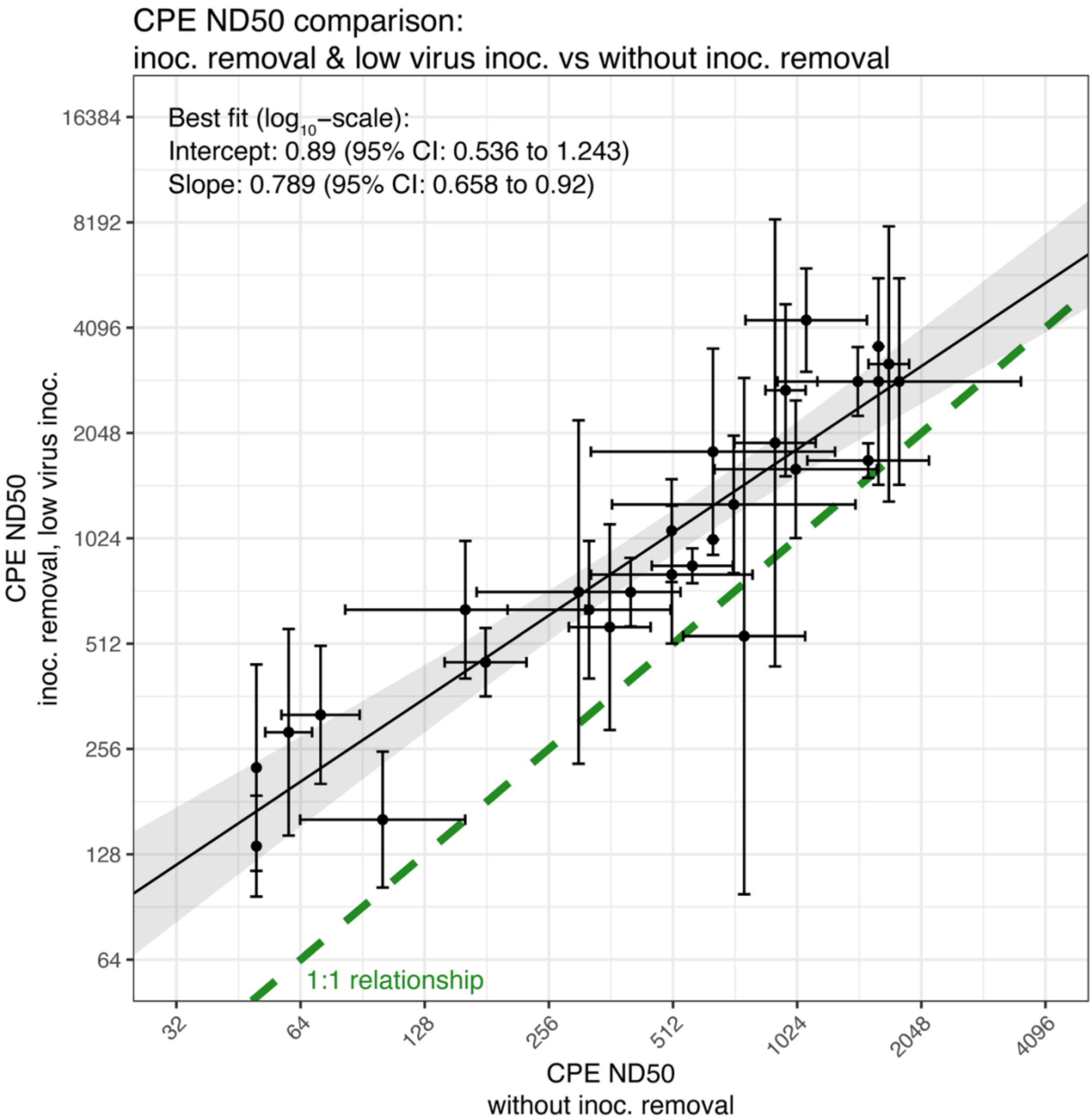

Fig. A 5 CPE ND50 comparison with and without removal of inoculum

Both CPE assays had the same virus inoculum (50 $TCID_{50}$). In the CPE assay with inoculum removal, the inoculum was replaced with fresh media after a 1-hour incubation (Materials and Methods). Removing the inoculum increased the ND50 (2.13-fold higher, 95% CI: 2.07-2.20, Table A 6) and back titration data indicates that removing the inoculum reduces the estimated number of infectious virions per well from 40 (95% CI: 18.1 to 62.0) to 3 (95% CI: 2.8 to 3.8, Table A 8).

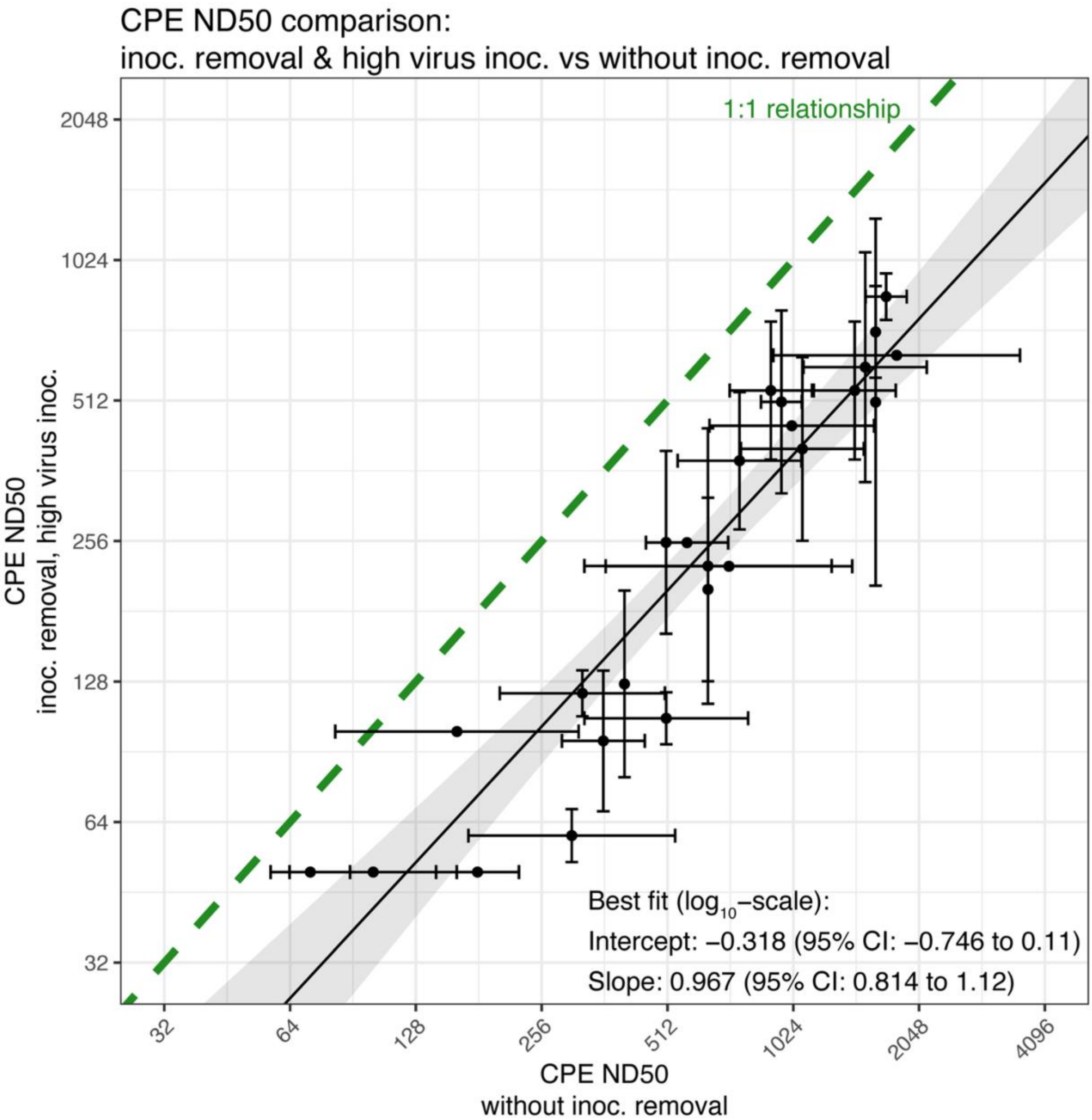

Fig. A 6 CPE ND50 comparison with and without removal of inoculum (comparable estimated number of infectious virions per well)

Both CPE assays had a comparable estimated number of infectious virions per well: on average 40 virions without inoculum removal and 35.5 virions with inoculum removal and higher virus inoculum (Table A 8). In the CPE assay with inoculum removal, the inoculum was replaced with fresh media after a 1-hour incubation (Materials and Methods). Removing the inoculum but maintaining the estimated number of infectious virions per well decreased the ND50 (2.57-fold lower, 95% CI: 2.51-2.62, Table A 6).

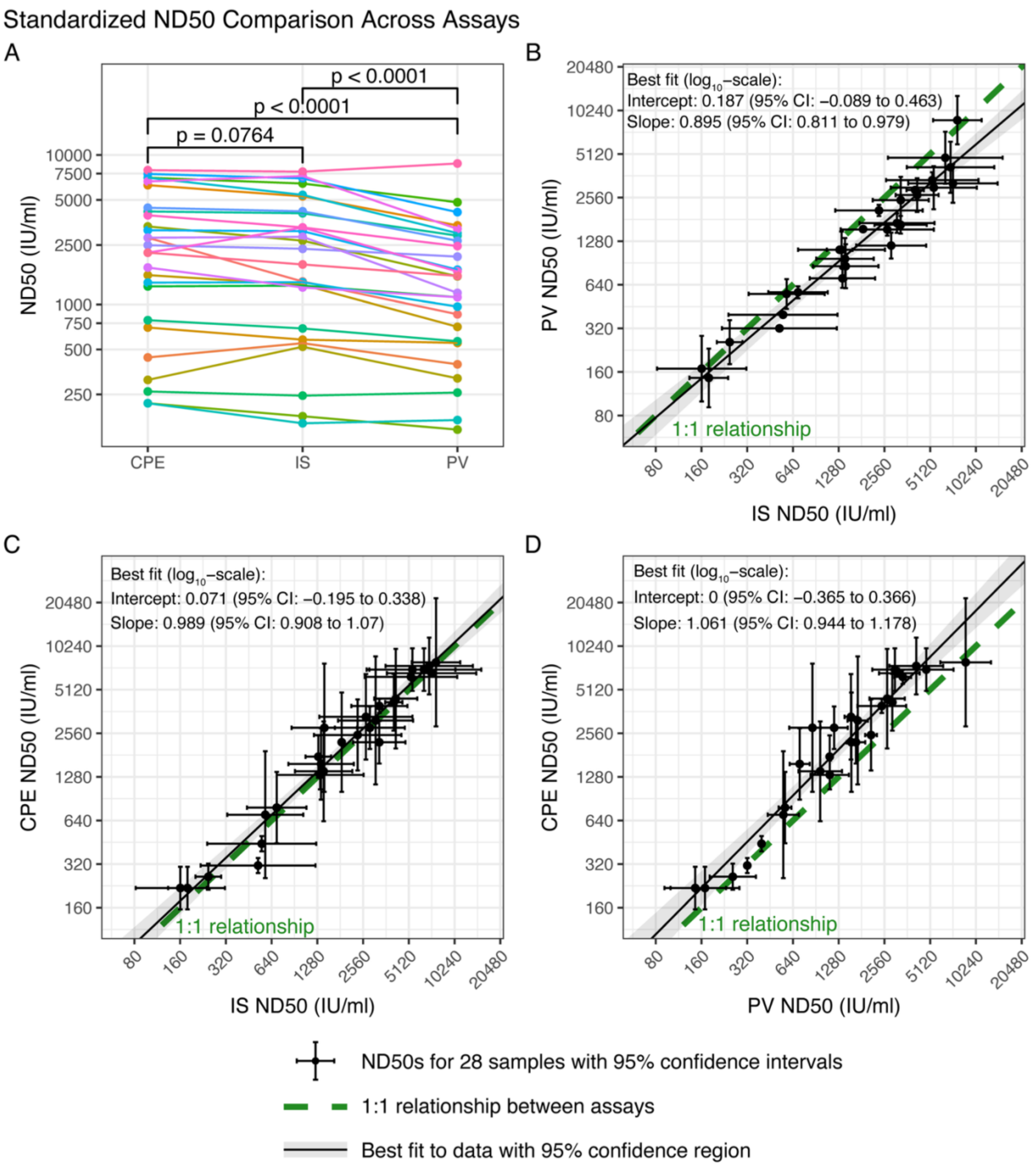

Fig. A 7 Comparison of normalized ND50s measured with IS, PV, and CPE assay

ND50s were normalized using the WHO standard NIBSC 21/338 (Tables A 1-3) and were compared using a paired t-test (A) and a linear fit to pairwise comparisons between ND50s (B-D). After normalization, there was no significant difference between CPE and IS assay ND50s (p = 0.076, paired t-test, panel A) but there was a significant difference between IS and PV ND50s and CPE and PV ND50s (A, B, D). Fold-differences between normalized ND50s are reported in Table A 7.

Tables

Table A 1 IS assay: Estimated ND50s

Sample	Sample name	Assay run 1 ND50	Assay run 2 ND50	ND50	ND50 95% CI	IU/ml	IU/ml 95% CI
1	High 3	1730.6	2127.0	1918.6	1567.5 - 2348.4	1422.9	865.6 - 2339
2	1678-044_pv2	792.2	696.0	742.6	654.1 - 843	550.7	241.1 - 1257.9
3	1678-044_PB1	7134.8	7124.3	7129.6	7119.3 - 7139.9	5287.4	2624.1 - 10653.9
4	1678-047_pv2	736.5	834.7	784.1	693.6 - 886.4	581.5	326.7 - 1035
5	1678-047_post_v3	1646.8	2026.5	1826.8	1490.7 - 2238.7	1354.8	825.1 - 2224.4
6	1678-061_pv2	767.1	644.3	703.0	592.5 - 834.1	521.4	218.4 - 1244.6
7	1678-061_post_v3	3614.2	3589.5	3601.8	3577.7 - 3626.1	2671.2	1318.7 - 5410.7
8	1678-065_pv2	196.1	296.7	241.2	160.7 - 361.9	178.9	133.4 - 239.9
9	1678-065_post_v3	9466.9	7961.7	8681.7	7326.7 - 10287.4	6438.5	2700.5 - 15350.5
10	1678-069 (008)_pv2	1764.5	1839.7	1801.7	1729.5 - 1876.9	1336.2	691.8 - 2580.7
11	1678-069 (008)_M9	255.6	427.9	330.7	199.6 - 548	245.3	202 - 297.9
12	1678-070 (043)_pv2	821.2	1058.3	932.3	727.1 - 1195.3	691.4	440.6 - 1085
13	1678-070 (043)_post_v3	4113.0	7346.3	5496.8	3113.5 - 9704.7	4076.5	3577 - 4645.8
14	1678-074_pv2	214.0	218.9	216.4	211.6 - 221.3	160.5	81.6 - 315.8
15	1678-074_post_v3	7113.8	7463.3	7286.5	6952 - 7637.1	5403.8	2814.9 - 10373.5
16	1678-097_pv2	1659.9	2172.7	1899.0	1458.7 - 2472.4	1408.4	911.3 - 2176.6
17	1678-097_post_v3	3903.5	4458.9	4172.0	3662 - 4753	3094.0	1751.9 - 5464.3
18	1678-044(101)_post_inf_visit	9224.8	9579.5	9400.5	9059.2 - 9754.5	6971.5	3595.4 - 13517.9
19	1678-061(117)_post_inf_visit	4681.9	6854.0	5664.8	3899.2 - 8229.8	4201.1	3033.4 - 5818.2
20	1678-066(122)_post_inf_visit	3119.2	3238.8	3178.4	3063.3 - 3297.9	2357.2	1215.5 - 4571
21	1678-070(126)_post_inf_visit	3507.7	4169.8	3824.4	3228.3 - 4530.6	2836.3	1669.9 - 4817.2
22	1678-078(037)_post_inf_visit	1353.8	2266.3	1751.6	1057.2 - 2902.1	1299.0	1069.7 - 1577.5
23	1678-090(144)_post_inf_visit	9636.9	9855.7	9745.7	9533.7 - 9962.4	7227.6	3672 - 14225.8
24	1678-091(145)_post_inf_visit	3779.4	5149.2	4411.5	3258.1 - 5973.3	3271.6	2201.7 - 4861.6
25	1678-095(149)_post_inf_visit	3841.4	5083.2	4418.9	3358.1 - 5814.7	3277.1	2143.2 - 5010.8
26	1678-099(153)_post_inf_visit	8800.2	12341.8	10421.6	7481.5 - 14517.2	7728.9	5350.9 - 11163.7
27	1678-SM_post_inf_visit	1777.4	3505.9	2496.3	1282.9 - 4857.3	1851.3	1790.3 - 1914.3
28	NIBSC 21/338[#]	4440.8	9063.6	6344.3	3153.1 - 12764.9	4705.0	

[#] Positive international reference plasma obtained from NIBSC.

Table A 2 PV assay: Estimated ND50s

Sample	Sample name	Assay run 1 ND50	Assay run 2 ND50	ND50	ND50 95% CI	IU/ml	IU/ml 95% CI
1	High 3	1670.2	1102.5	1356.9	903.2 - 2038.7	859.7	605.4 - 1220.9
2	1678-044_pv2	639.4	616.3	627.7	605.5 - 650.8	397.7	389.7 - 405.8
3	1678-044_PB1	5151.2	5528.6	5336.6	4979.4 - 5719.4	3381.2	2982.1 - 3833.7
4	1678-047_pv2	796.4	957.0	873.0	729.1 - 1045.3	553.1	436.7 - 700.6
5	1678-047_post_v3	1065.4	1174.2	1118.5	1016.8 - 1230.3	708.6	608.9 - 824.7
6	1678-061_pv2	527.8	484.4	505.7	464.9 - 550	320.4	311.6 - 329.4
7	1678-061_post_v3	2631.4	2250.1	2433.3	2087.2 - 2836.8	1541.7	1399 - 1698.9
8	1678-065_pv2	186.3	283.1	229.7	152.5 - 346.1	145.5	91.3 - 232
9	1678-065_post_v3	9678.1	5993.5	7616.2	4762 - 12180.9	4825.5	3192 - 7295
10	1678-069 (008)_pv2	1566.4	1987.2	1764.3	1397.4 - 2227.6	1117.9	836.9 - 1493.2
11	1678-069 (008)_M9	349.2	471.5	405.8	302.3 - 544.7	257.1	181.1 - 365.1
12	1678-070 (043)_pv2	877.7	914.0	895.7	860.9 - 931.9	567.5	515.6 - 624.6
13	1678-070 (043)_post_v3	4714.1	4402.6	4555.7	4260.5 - 4871.4	2886.4	2855.8 - 2917.4
14	1678-074_pv2	210.0	337.6	266.2	167.2 - 424	168.7	100.1 - 284.2
15	1678-074_post_v3	5800.8	3860.7	4732.3	3175.4 - 7052.7	2998.3	2128.4 - 4223.8
16	1678-097_pv2	1327.6	1756.2	1526.9	1160.7 - 2008.6	967.4	695.2 - 1346.4
17	1678-097_post_v3	3002.3	2420.6	2695.8	2182.9 - 3329.3	1708.0	1463.2 - 1993.8
18	1678-044(101)_post_inf_visit	8292.5	5168.8	6546.9	4119.6 - 10404.5	4148.0	2761.3 - 6231.1
19	1678-061(117)_post_inf_visit	4966.8	3567.9	4209.6	3044.1 - 5821.4	2667.1	2040.4 - 3486.4
20	1678-066(122)_post_inf_visit	3552.6	3061.8	3298.1	2851 - 3815.4	2089.6	1911 - 2285
21	1678-070(126)_post_inf_visit	1746.4	2035.1	1885.2	1622.7 - 2190.1	1194.4	971.8 - 1468
22	1678-078(037)_post_inf_visit	1822.1	1706.0	1763.1	1652.9 - 1880.5	1117.0	1107.9 - 1126.2
23	1678-090(144)_post_inf_visit	6095.8	4204.1	5062.3	3517.4 - 7285.9	3207.4	2357.7 - 4363.4
24	1678-091(145)_post_inf_visit	2514.4	2740.4	2625.0	2412.7 - 2856	1663.2	1444.9 - 1914.3
25	1678-095(149)_post_inf_visit	4823.6	3123.4	3881.5	2535.3 - 5942.5	2459.3	1699.4 - 3558.9
26	1678-099(153)_post_inf_visit	17327.8	11057.9	13842.3	8913.3 - 21496.8	8770.2	5974.5 - 12874.2
27	1678-SM_post_inf_visit	2494.0	2404.5	2448.9	2362.7 - 2538.1	1551.6	1520.1 - 1583.7
28	NIBSC 21/338[#]	7642.5	7215.7	7426.0	7019.3 - 7856.3	4705.0	

[#] Positive international reference plasma obtained from NIBSC.

Table A 3 CPE assay: Estimated ND50s

Sample	Sample name	Assay run 1 ND50	Assay run 2 ND50	ND50	ND50 95% CI	IU/ml	IU/ml 95% CI
1	High 3	452.0	906.0	639.9	323.7 - 1265	2795.9	1008.1 - 7754.4
2	1678-044_pv2	128.0	80.0	101.2	63.8 - 160.4	442.1	391.4 - 499.5
3	1678-044_PB1	1612.0	1280.0	1436.4	1145.9 - 1800.7	6275.9	5607.3 - 7024.3
4	1678-047_pv2	114.0	226.0	160.5	82.1 - 313.9	701.3	255.6 - 1924.1
5	1678-047_post_v3	320.0	404.0	359.6	286.1 - 451.8	1570.9	891 - 2769.7
6	1678-061_pv2	80.0	64.0	71.6	57.5 - 89	312.6	277.3 - 352.5
7	1678-061_post_v3	640.0	906.0	761.5	541.7 - 1070.5	3326.9	1686.7 - 6562.2
8	1678-065_pv2	50.0	50.0	50.0	50 - 50	218.5	155.7 - 306.5
9	1678-065_post_v3	1612.0	1612.0	1612.0	1612 - 1612	7043.0	5019.7 - 9881.7
10	1678-069 (008)_pv2	404.0	226.0	302.2	171 - 533.9	1320.2	1048.3 - 1662.6
11	1678-069 (008)_M9	64.0	56.0	59.9	52.5 - 68.2	261.6	212.5 - 322
12	1678-070 (043)_pv2	160.0	202.0	179.8	143.1 - 225.9	785.5	445.5 - 1384.9
13	1678-070 (043)_post_v3	906.0	1016.0	959.4	857.5 - 1073.4	4191.8	2670.3 - 6580.3
14	1678-074_pv2	50.0	50.0	50.0	50 - 50	218.5	155.7 - 306.5
15	1678-074_post_v3	1612.0	1612.0	1612.0	1612 - 1612	7043.0	5019.7 - 9881.7
16	1678-097_pv2	254.0	404.0	320.3	203.3 - 504.8	1399.6	633 - 3094.5
17	1678-097_post_v3	508.0	1016.0	718.4	364.2 - 1417	3138.8	1134.2 - 8686.6
18	1678-044(101)_post_inf_visit	1612.0	1810.0	1708.1	1524.8 - 1913.5	7463.0	4748.2 - 11729.9
19	1678-061(117)_post_inf_visit	806.0	1280.0	1015.7	645.5 - 1598.2	4437.8	2010.2 - 9797
20	1678-066(122)_post_inf_visit	508.0	640.0	570.2	454.7 - 715	2491.2	1415.9 - 4383.3
21	1678-070(126)_post_inf_visit	640.0	640.0	640.0	640 - 640	2796.2	1992.9 - 3923.3
22	1678-078(037)_post_inf_visit	404.0	404.0	404.0	404 - 404	1765.1	1258 - 2476.6
23	1678-090(144)_post_inf_visit	1810.0	1280.0	1522.1	1083.9 - 2137.5	6650.2	6644.4 - 6656
24	1678-091(145)_post_inf_visit	508.0	508.0	508.0	508 - 508	2219.5	1581.9 - 3114.1
25	1678-095(149)_post_inf_visit	1016.0	806.0	904.9	721.2 - 1135.4	3953.7	3535.7 - 4421.2
26	1678-099(153)_post_inf_visit	1280.0	2560.0	1810.2	917.7 - 3570.5	7908.9	2857.8 - 21887.5
27	1678-SM_post_inf_visit	404.0	640.0	508.5	324 - 798.1	2221.6	1008.8 - 4892.7
28	NIBSC 21/338#	1280.0	906.0	1076.9	767.5 - 1510.9	4705.0	4705 - 4705

Positive international reference plasma obtained from NIBSC.

Table A 4 CPE assay (with inoculum removal, lower virus inoculum): Estimated ND50s

Sample	Sample name	Assay run 1 ND50	Assay run 2 ND50	ND50	ND50 95% CI	IU/ml	IU/ml 95% CI
1	High 3	2560.0	1280.0	1810.2	917.7 - 3570.5	1978.3	714.1 - 5480.8
2	1678-044_pv2	202.0	128.0	160.8	102.8 - 251.5	175.7	80 - 386
3	1678-044_PB1	3226.0	2560.0	2873.8	2291.1 - 3604.7	3140.7	1782.6 - 5533.3
4	1678-047_pv2	508.0	806.0	639.9	407 - 1005.9	699.3	624.8 - 782.7
5	1678-047_post_v3	806.0	404.0	570.6	290 - 1122.8	623.6	225.6 - 1723.6
6	1678-061_pv2	404.0	254.0	320.3	203.3 - 504.8	350.1	158.2 - 774.9
7	1678-061_post_v3	1280.0	226.0	537.8	98.3 - 2942.3	587.8	76.5 - 4516.5
8	1678-065_pv2	320.0	160.0	226.3	114.7 - 446.3	247.3	89.3 - 685.1
9	1678-065_post_v3	2032.0	4064.0	2873.7	1456.9 - 5668.2	3140.6	2236.4 - 4410.3
10	1678-069 (008)_pv2	404.0	1280.0	719.1	232.3 - 2226.4	785.9	356.5 - 1732.3
11	1678-069 (008)_M9	202.0	404.0	285.7	144.8 - 563.5	312.2	222.3 - 438.4
12	1678-070 (043)_pv2	404.0	508.0	453.0	361.9 - 567	495.1	441.2 - 555.6
13	1678-070 (043)_post_v3	2032.0	3620.0	2712.2	1540.1 - 4776.2	2964.1	2364.1 - 3716.2
14	1678-074_pv2	114.0	160.0	135.1	96.9 - 188.3	147.6	146.5 - 148.7
15	1678-074_post_v3	3620.0	3620.0	3620.0	3620 - 3620	3956.2	2816.6 - 5556.8
16	1678-097_pv2	806.0	508.0	639.9	407 - 1005.9	699.3	316.7 - 1544.1
17	1678-097_post_v3	1016.0	1612.0	1279.8	814.1 - 2011.8	1398.6	1249.7 - 1565.3
18	1678-044(101)_post_inf_visit	2032.0	5120.0	3225.5	1304 - 7978.3	3525.1	2001.7 - 6207.7
19	1678-061(117)_post_inf_visit	1280.0	2032.0	1612.7	1025.3 - 2536.7	1762.5	1573.9 - 1973.7
20	1678-066(122)_post_inf_visit	906.0	806.0	854.5	762 - 958.3	933.9	592.9 - 1471
21	1678-070(126)_post_inf_visit	1016.0	1016.0	1016.0	1016 - 1016	1110.4	790.5 - 1559.6
22	1678-078(037)_post_inf_visit	640.0	806.0	718.2	572.9 - 900.3	784.9	700.5 - 879.5
23	1678-090(144)_post_inf_visit	1810.0	1612.0	1708.1	1524.8 - 1913.5	1866.8	1186.4 - 2937.3
24	1678-091(145)_post_inf_visit	906.0	1280.0	1076.9	767.5 - 1510.9	1176.9	1175.6 - 1178.2
25	1678-095(149)_post_inf_visit	906.0	4064.0	1918.8	440.8 - 8352.5	2097.1	676.7 - 6498.9
26	1678-099(153)_post_inf_visit	2032.0	4064.0	2873.7	1456.9 - 5668.2	3140.6	2236.4 - 4410.3
27	1678-SM_post_inf_visit	640.0	1016.0	806.4	512.7 - 1268.3	881.3	787 - 986.9
28	NIBSC 21/338[#]	3620.0	5120.0	4305.2	3065.1 - 6047	4705.0	4705 - 4705

[#] Positive international reference plasma obtained from NIBSC.

Table A 5 CPE assay (with inoculum removal, higher virus inoculum): Estimated ND50s

Sample	Sample name	Assay run 1 ND50	Assay run 2 ND50	ND50	ND50 95% CI	IU/ml	IU/ml 95% CI
1	High 3	320.0	160.0	226.3	114.7 - 446.3	2640.5	851.1 - 8192
2	1678-044_pv2	50.0	Below LOD	50.0		735.2	
3	1678-044_PB1	640.0	452.0	537.8	382.5 - 756.3	6276.4	2837.9 - 13881.2
4	1678-047_pv2	100.0	Below LOD	100.0		1470.3	
5	1678-047_post_v3	114.0	80.0	95.5	67.5 - 135.1	1114.4	500.7 - 2480.2
6	1678-061_pv2	50.0	Below LOD	50.0		735.2	
7	1678-061_post_v3	452.0	320.0	380.3	271.1 - 533.5	4438.1	2011.5 - 9792.2
8	1678-065_pv2	Below LOD	Below LOD	Below LOD		Below LOD	
9	1678-065_post_v3	806.0	640.0	718.2	572.9 - 900.3	8381.3	4250.7 - 16525.6
10	1678-069 (008)_pv2	64.0	56.0	59.9	52.5 - 68.2	698.6	389.7 - 1252.5
11	1678-069 (008)_M9	Below LOD	Below LOD	Below LOD		Below LOD	
12	1678-070 (043)_pv2	50.0	50.0	50.0	50 - 50	583.5	371 - 917.7
13	1678-070 (043)_post_v3	640.0	404.0	508.5	324 - 798.1	5933.8	2403.5 - 14649.7
14	1678-074_pv2	Below LOD	Below LOD	Below LOD		Below LOD	
15	1678-074_post_v3	806.0	320.0	507.9	205.4 - 1255.7	5926.5	1523.9 - 23048.6
16	1678-097_pv2	114.0	128.0	120.8	107.8 - 135.3	1409.6	1004 - 1979.3
17	1678-097_post_v3	226.0	226.0	226.0	226 - 226	2637.3	1676.7 - 4148.2
18	1678-044(101)_post_inf_visit	906.0	806.0	854.5	762 - 958.3	9972.0	5653.4 - 17589.6
19	1678-061(117)_post_inf_visit	452.0	452.0	452.0	452 - 452	5274.6	3353.5 - 8296.3
20	1678-066(122)_post_inf_visit	254.0	254.0	254.0	254 - 254	2964.1	1884.5 - 4662.1
21	1678-070(126)_post_inf_visit	254.0	160.0	201.6	128.2 - 317.1	2352.5	950.9 - 5820
22	1678-078(037)_post_inf_visit	100.0	160.0	126.5	79.8 - 200.5	1476.1	1464.8 - 1487.5
23	1678-090(144)_post_inf_visit	806.0	452.0	603.6	342.4 - 1063.9	7043.5	2540.5 - 19527.8
24	1678-091(145)_post_inf_visit	320.0	202.0	254.2	162 - 399.1	2966.9	1201.7 - 7324.8
25	1678-095(149)_post_inf_visit	640.0	452.0	537.8	382.5 - 756.3	6276.4	2837.9 - 13881.2
26	1678-099(153)_post_inf_visit	640.0	640.0	640.0	640 - 640	7468.5	4748.3 - 11747
27	1678-SM_post_inf_visit	114.0	100.0	106.8	93.9 - 121.4	1246.0	696.7 - 2228.3
28	NIBSC 21/338[#]	320.0	508.0	403.2	256.3 - 634.2	4705.0	4705 - 4705

[#] Positive international reference plasma obtained from NIBSC.

Table A 6 Mean fold-difference of ND50s between assays

	IS assay	PV assay	CPE assay	CPE assay (inoculum removal, lower inoculum)	CPE assay (inoculum removal, higher inoculum)
**IS assay**		1.2 (1.18 - 1.23)	5.46 (5.37 - 5.54)	2.56 (2.47 - 2.65)	14.2 (13.88 - 14.52)
**PV assay**	0.83 (0.81 - 0.85)		4.53 (4.43 - 4.63)	2.12 (2.06 - 2.19)	11.36 (11.04 - 11.68)
**CPE assay**	0.18 (0.18 - 0.19)	0.22 (0.22 - 0.23)		0.47 (0.45 - 0.48)	2.57 (2.51 - 2.62)
**CPE assay (inoculum removal, lower inoculum)**	0.39 (0.38 - 0.4)	0.47 (0.46 - 0.49)	2.13 (2.07 - 2.20)		5.09 (4.93 - 5.26)
**CPE assay (inoculum removal, higher inoculum)**	0.07 (0.07 - 0.07)	0.09 (0.09 - 0.09)	0.39 (0.38 - 0.40)	0.20 (0.19 - 0.20)	

Mean fold-difference of ND50s of 28 samples measured with different assays (with 95% CIs). The fold-differences are for rows relative to columns (i.e. the first row are the IS assay ND50s relative to the PV assay ND50s, CPE assay ND50s, etc.). ND50s were not normalized to IU/ml for this assay comparison.

Table A 7 Mean fold-difference of normalized ND50s between assays

	IS assay	PV assay	CPE assay	CPE assay (inoculum removal, lower inoculum)	CPE assay (inoculum removal, higher inoculum)
**IS assay**		1.43 (1.40 - 1.46)	0.92 (0.91 - 0.94)	1.77 (1.71 - 1.83)	0.87 (0.85 - 0.90)
**PV assay**	0.70 (0.69 - 0.71)		0.65 (0.63 - 0.66)	1.24 (1.20 - 1.28)	0.59 (0.57 - 0.60)
**CPE assay**	1.08 (1.07 - 1.10)	1.55 (1.51 - 1.58)		1.92 (1.86 - 1.98)	0.93 (0.91 - 0.96)
**CPE assay (inoculum removal, lower inoculum)**	0.56 (0.55 - 0.58)	0.81 (0.78 - 0.83)	0.52 (0.51 - 0.54)		0.45 (0.44 - 0.46)
**CPE assay (inoculum removal, higher inoculum)**	1.15 (1.12 - 1.18)	1.70 (1.65 - 1.75)	1.07 (1.04 - 1.10)	2.23 (2.15 - 2.30)	

Mean fold-difference of normalized ND50s (in IU/ml) of 28 samples measured with different assays (with 95% CIs). The fold-differences are for rows relative to columns (i.e. the first row contains the normalized IS assay ND50s relative to the normalized PV assay ND50s, CPE assay ND50s, etc.).

Table A 8 Estimated number of infectious virions per well in CPE assays

Data	Estimated infectious virions per well	95% confidence interval
Original CPE (without inoculum removal)	40.03	18.06 to 62.00
CPE (with inoculum removal, lower virus inoculum)	3.32	2.84 to 3.79
CPE (with inoculum removal, higher virus inoculum)	35.47	21.42 to 49.52

Table A 9 Observed and predicted relationships between CPE and IS assay ND50s

Parameter (for ND50 comparison on a $\log_{10}$-scale)	Observed relationship (best fit to ND50s)	Predicted relationship (based on 'per well' vs 'per virion' assay format)
CPE assay without inoculum removal vs IS assay (Fig. 1C and Fig. 3A)		
Intercept (95% confidence interval)	-0.692 (-0.961 to -0.423)	-1.416 (-1.582 to -1.129)
Slope (95% confidence interval)	0.987 (0.908 to 1.065)	1
CPE assay with inoculum removal & low virus inoculum vs without inoculum removal (Fig. A 5)		
Intercept (95% confidence interval)	0.890 (0.536 to 1.243)	-
Slope (95% confidence interval)	0.789 (0.658 to 0.920)	-
CPE assay with inoculum removal & high virus inoculum vs without inoculum removal (Fig. A 6)		
Intercept (95% confidence interval)	-0.318 (-0.746 to 0.110)	-
Slope (95% confidence interval)	0.967 (0.814 to 1.120)	-
CPE assay with removal of inoculum & low virus inoculum vs IS assay (Fig. 3C)		
Intercept (95% confidence interval)	0.351 (-0.154 to 0.856)	-0.511 (-0.566 to -0.448)
Slope (95% confidence interval)	0.776 (0.629 to 0.924)	1
CPE assay with removal of inoculum & high virus inoculum vs IS assay (Fig. 3B)		
Intercept (95% confidence interval)	-1.237 (-1.820 to -0.654)	-1.374 (-1.505 to -1.191)
Slope (95% confidence interval)	1.024 (0.859 to 1.189)	1
